# Immunobiochemical Reconstruction of Influenza Lung Infection—Melanoma Skin Cancer Interactions

**DOI:** 10.3389/fimmu.2019.00004

**Published:** 2019-01-28

**Authors:** Evgeni V. Nikolaev, Andrew Zloza, Eduardo D. Sontag

**Affiliations:** ^1^Center for Quantitative Biology, Rutgers University, Piscataway, NJ, United States; ^2^Clinical Investigations and Precision Therapeutics Program, Rutgers Cancer Institute of New Jersey, New Brunswick, NJ, United States; ^3^Section of Surgical Oncology Research, Division of Surgical Oncology, Rutgers Cancer Institute of New Jersey, New Brunswick, NJ, United States; ^4^Department of Surgery, Rutgers Robert Wood Johnson Medical School, New Brunswick, NJ, United States; ^5^Department of Electrical and Computer Engineering, Northeastern University, Boston, MA, United States; ^6^Department of Bioengineering, Northeastern University, Boston, MA, United States; ^7^Laboratory for Systems Pharmacology, Harvard Medical School, Boston, MA, United States

**Keywords:** influenza, melanoma, PD-1/PD-L1, incoherent feedforward loop, mathematical modeling

## Abstract

It was recently reported that acute influenza infection of the lung promoted distal melanoma growth in the dermis of mice. Melanoma-specific CD8+ T cells were shunted to the lung in the presence of the infection, where they expressed high levels of inflammation-induced cell-activation blocker PD-1, and became incapable of migrating back to the tumor site. At the same time, co-infection virus-specific CD8+ T cells remained functional while the infection was cleared. It was also unexpectedly found that PD-1 blockade immunotherapy reversed this effect. Here, we proceed to ground the experimental observations in a mechanistic immunobiochemical model that incorporates T cell pathways that control PD-1 expression. A core component of our model is a kinetic motif, which we call a PD-1 Double Incoherent Feed-Forward Loop (DIFFL), and which reflects known interactions between IRF4, Blimp-1, and Bcl-6. The different activity levels of the PD-1 DIFFL components, as a function of the cognate antigen levels and the given inflammation context, manifest themselves in phenotypically distinct outcomes. Collectively, the model allowed us to put forward a few working hypotheses as follows: (i) the melanoma-specific CD8+ T cells re-circulating with the blood flow enter the lung where they express high levels of inflammation-induced cell-activation blocker PD-1 in the presence of infection; (ii) when PD-1 receptors interact with abundant PD-L1, constitutively expressed in the lung, T cells loose motility; (iii) at the same time, virus-specific cells adapt to strong stimulation by their cognate antigen by lowering the transiently-elevated expression of PD-1, remaining functional and mobile in the inflamed lung, while the infection is cleared. The role that T cell receptor (TCR) activation and feedback loops play in the underlying processes are also highlighted and discussed. We hope that the results reported in our study could potentially contribute to the advancement of immunological approaches to cancer treatment and, as well, to a better understanding of a broader complexity of fundamental interactions between pathogens and tumors.

## 1. Introduction

It was recently reported that acute influenza A infection (A/H1N1/PR8) of the lung promoted distal B16-F10 skin melanoma growth in the dermis (1). It was also observed that melanoma-specific CD8+ T cells were shunted to the lung in the presence of the infection, where they expressed high levels of inflammation-induced cell-activation blocker PD-1, and became incapable of migrating back to the tumor site. At the same time, co-infection virus-specific CD8+ T cells remained functional while the infection was cleared. Finally, it was also unexpectedly found that blockade of PD-1 resulted in reversal of infection-mediated anti-tumor response disruption.

In this respect, it is very important to mention that the work by Kohlhapp et al. (1) was primarily motivated by two still unmet challenges: (i) emerging epidemiological studies reporting an increased cancer prevalence and cancer-specific deaths in patients with infections (1), and (ii) despite the fact that tremendous amount of work on immune response in the context of pathogenic co-infection has been done, findings in this field still remain discordant and a matter of debate, as also reviewed by Kohlhapp et al. (1) and Zloza (2).

Motivated by the need to provide a more conceptual and quantitative biology insight into “the previously unrecognized acute non-oncogenic infection factor” accelerating tumor growth (1) and more broadly into the interactions between pathogens and cancer, and specifically, in order “to harness these interactions to improve microbial-based cancer therapy” (2), we suggest a few immunobiochemical mechanisms and a simple mathematical model which may help to interpret the observed phenomena (1).

Our main results relate to two fundamental functional roles of immunity (3–5): (i) adaptation of immune function, and (ii) competition between excitation and de-excitation (“push-pull”) factors possessing different response kinetics. In the context of this work, the loss of adaptation occurs in the expression of PD-1 receptors on anti-melanoma CD8+ T cells, a phenomenon that may constitute the essence of the previously unrecognized immunologic factor (1), while competing push-pull factors (3) correspond to opposite outcomes of the corresponding kinetic motifs identified as incoherent feedforward loops (IFFLs) in the classification of Alon (6). We briefly note that push-pull factors also play multiple fundamental roles in physiology (and biology) in general, e.g., Dampney et al. (7).

Our working hypothesis is that the melanoma-specific T cells shunted to the lung in the presence of the infection express high levels of inflammation-induced cell-activation blocker PD-1, which upon interacting with PD-L1 constitutively expressed in the lung, render T cell motility paralysis (8). At the same time, virus-specific cells adapt to strong stimulation by their cognate antigen by lowering the transiently-elevated expression of PD-1, remaining functional and mobile while the infection is cleared.

Although other important mechanisms may contribute to the previously unrecognized acute non-oncogenic infection factor (1), we focus our efforts on one concrete aspect of the problem, which is a gene regulatory network (GRN) that controls PD-1 expression. Indeed, the fact that many other factors may contribute to the enormously complex molecular makeup of the acute non-oncogenic infection effect, such factors, obviously, do not exclude the interaction PD-1:PD-L1 playing a role as clearly seen from the data collected in Kohlhapp et al. (1). Thus, the importance of the PD-1:PD-L1 signal sent by the data cannot be disputed. Moreover, it is the PD-1:PD-L1 signal “detected” experimentally in Kohlhapp et al. (1) that defines the scope of our work aimed in uncovering relevant molecular detail in an unbiased way. We then develop and use a simple mathematical model in order to further illuminate the PD-1:PD-L1 role.

Specifically, a core component of our PD-1 gene-regulatory network (GRN) is a kinetic motif, which we call a Double Incoherent Feed-Forward Loop (PD-1 DIFFL), and which reflects known interactions between IRF4, Blimp-1, and Bcl-6 transcription factors (TFs). The different activity levels of the PD-1 DIFFL components, as a function of (a) the cognate antigen levels, (b) the T cell receptor (TCR) activity, and (c) the given inflammation context, manifest themselves in the T cell phenotypically distinct outcomes discussed in our work.

The rest of our work is organized as follows. In section 2.1, the main results of Kohlhapp et al. (1) are briefly outlined. Alternative hypotheses potentially related to the unrecognized factor are discussed in section 2.2. Here, the motivation for the development of the PD-1 DIFFL is also given. The PD-1 DIFFL is reconstructed in section 2.3. We next attempt to falsify and validate the kinetic motif (PD-1 DIFFL) against key experiential observations in section 2.4. The results of our mathematical modeling are described in section 2.5. Finally, a literature review of the corresponding mechanistic detail, the model construction, and the model's parameter justification can be found in Supplementary Material.

## 2. Results

We begin our analysis of the experimental data (1) by discussing a few alternative hypotheses, followed by the introduction of a number of mechanisms consistent with the discussed observations.

The selected mechanisms will then be formalized in terms of a relevant genetic molecular circuit (PD-1 DIFFL) that regulates PD-1 expression. Our proposed PD-1 DIFFL model is based upon molecular detail discovered previously, and is independent of the results obtained in Kohlhapp et al. (1).

We hope that the strong inference methodological approach (9) that guides our research will allow us to customize the PD-1 DIFFL to different inflammatory conditions (1) with the ultimate goal to capture both infection-tumor and infection-infection interactions at the mechanistic molecular level.

### 2.1. Linking Observations With Immunological Mechanisms

A key challenge in the study of T cells within different dual immunological self (tumor) and non-self (infectious) contexts, is the organization of large amounts of relevant molecular and biochemical information (section SI-1) compactly summarized in Table 1.

**Table 1:** A summary of the immunological reconstruction of infection-tumor interactions^a^.

**Observation**	**Description**	**Mechanism (hypothesis)**
**(O1)** Anti-tumor CD8+ T cells are shunted to the infected site.	Tumor-specific CD8+ T cells of infected hosts were significantly reduced on day 6 in the TME compared to uninfected hosts and found at high levels at the site of infection but not observed in tissues unrelated to the tumor challenge or infection.	**(O1-M1)** Low-affinity immunological synapses formed between TCRs on anti-tumor CD8+ T cells and self-antigens on tumor cells lead to the lack of the Ag-induced arrest of the anti-tumor CD8+ T cells in the TME.
		**(O1-M2)** Infection-induced chemokines and cytokines amplify the tumor's ability to egress anti-tumor T_EFF_ from the TME.
		**(O1-M3)** Non-specific cardiovascular edema caused by infection-induced inflammation affects anti-tumor T_EFF_ trafficking.
		**(O1-M4)** Infection-induced chemokines chemoattract anti-tumor T_EFF_ to the infected lung.
		**(O1-M5)** Infection-induced IL-2 retains all types of T_EFF_ in the infected lung.
		**(O1-M6)** Infection-induced cytokines amplify expression of endothelial PD-L1, which in turn leads to paralysis of anti-tumor T_EFF_ in the inflamed lung due to PD-1:PD-L1 bonds.
**(O2)** Cancer does not suppress the immune system anti-viral response which is capable at the same time of eradicating concomitant infections efficiently.	Cancer does not alter significantly the natural clearance of infection. Influenza infection also does not alter the natural clearance of VACV or the proportion of VACV-tetramer+ CD8+ T cells at the site of influenza infection.	**(O2-M1)** High-affinity immunological synapses formed between TCRs on anti-infection CD8+ T cells and nonself-antigens on infected cells lead to the Ag-induced arrest of the anti-infection CD8+ T cells inside the infected sites until a full clearance of the infection antigen.
**(O3)** Therapeutic PD-1 blockade reverses infection-mediated anti-tumor response disruption.	PD-1 blockade decelerates tumor growth in influenza-infected mice as well as rescues the percentage of anti-tumor CD8+ T cells within the TME.	**(O3-M1)** αPD-1 blockade shifts the dynamic equilibrium of the dynamically formed PD-1:PD-L1 bonds toward unbound forms of both PD-L1 and PD-1, allowing anti-tumor T_EFF_ to recover from immunologic paralysis and to gain motility.
		**(O3-M2)** Due to αPD-1 blockade, reactivated anti-tumor CD8+ T cells may recirculate back to the TME with constitutive lymph motion.
		**(O3-M3)** αPD-1 blockade reactivates PD-1-blocked signaling pathways leading to (i) improved killing capability, (ii) proliferation, (iii) suppression of PD-1 expression, (iv) protection against exhaustion, etc. in reactivated CD8+ T cells.

a *Literature citations are directly inserted through the text (section SI-1)*.

Specifically, Table 1 highlights the following key observations (O1)–(O5) made in Kohlhapp et al. (1):

**(O1)** *Distant influenza-melanoma interaction:* Influenza-induced loss of anti-melanoma CD8+ T cells from the tumor micro-environment (TME) and their sequestration in the infected lung.
**(O2)** *The host immune system's ability to respond to concomitant infection challenges:* influenza A virus (IAV) infection does not impede clearance of vaccinia virus (VACV) infection under the same conditions, nor tumor challenge changes the ability of the immune system to eliminate the infection.
**(O3)** *Reactivation of exhausted (*T_EX_*) anti-melanoma CD8+ T cells after anti-PD-1 (*α*PD-1) blockade:* (i) reactivated anti-melanoma CD8+ T cells which continue to reside in the TME regain their ability to contribute to the anti-tumor immune response and, additionally, (ii) reactivated anti-melanoma CD8+ T cells sequestered in the infected lung may regain their motility and return back to the TME, where they also aid in the anti-tumor response.
**(O4)** *Reduced host survival:* Infection early in tumor formation reduces host survival by promoting tumor growth in the infected host.
**(O5)** *Differential expression of PD-1 receptors by effector cells (*T_EFF_*) in the infected lung:* Anti-melanoma CD8+ T cells express larger amounts of PD-1 receptors than anti-influenza CD8+ T cells under the same conditions in the infected lung.

### 2.2. From a Physiologic Systemic View of Lymphocyte Re-circulation to Systems Biology of PD1:PD-L1 Interactions

It is known (10, 11) that non-specific cardiovascular edema effects, caused by infection-induced inflammation in the infected site, redirect the blood-flow to the site of infection-induced inflammation. Therefore, it is highly appealing to explain the observed accumulation of anti-melanoma CD8+ T cells in the infected lungs, (O1), by non-specific inflammation effects only.

**Figure 1:**
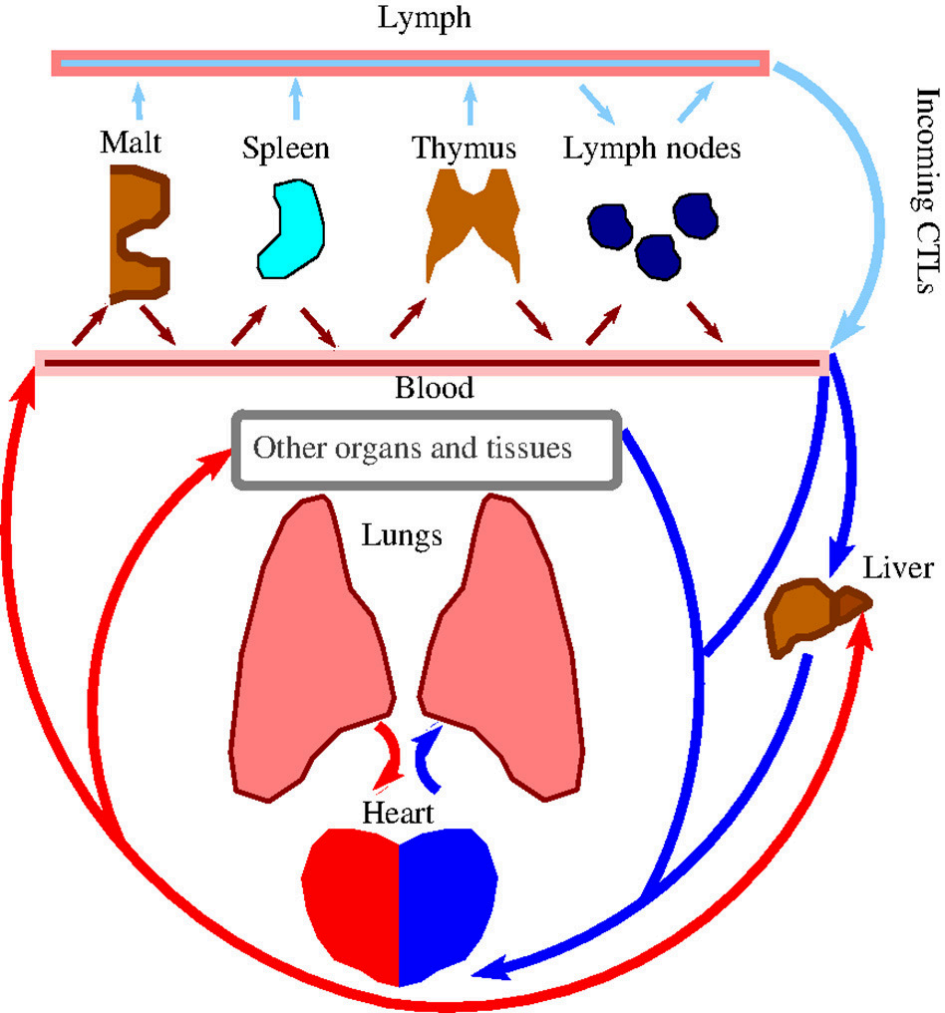
Schematic representation of lymphocyte re-circulation routes. There are two different routes for naïve and activated trafficking lymphocytes (12, 13). First, due to the data discussed in Owen et al. (12, Ch.14) and, independently estimated in Van den Berg (14, p. 23) after approximately 30 min. transit time in the blood, about 45% of all naïve lymphocytes (produced by the thymus and bone marrow) migrate to the spleen, where they reside for about 5 h. Another 45% of lymphocytes enter various peripheral nodes, where they remain for 12–18 h, scanning stromal cell surfaces. A smaller fraction of lymphocytes migrate to secondary lymphoid tissues (skin, gastrintestinal, etc.), to protect the organism against the external environment. Thus, about 5% of the lymphocytes are, at resting conditions, in the blood, and the majority resides in the lymph nodes. Second, as discussed in Poleszczuk et al. (15) activated CTLs enter the blood system via the great veins, flow through the pulmonary circulation, and, then, continue into systemic circulation. Venus blood from gastrointestinal tract and spleen goes to the liver through the hepatic portal vein. In all cases, lymphocytes migrate from the blood into lymph nodes through high-endothelial venues, specialized areas in postcappillary venues. (a) MALT is Mucosa Associated Lymphoid Tissue. (b) Lymph nodes have both afferent and efferent lymphatic vessels, while MALT, Spleen, and Thymus have efferent lymphatic only (12).

Note that the lymphocyte recirculation routes are phenotype-dependent and significantly differ for naïve/memory/effector subsets (12). We leave the corresponding details specific to the different subsets out of the discussion that follows. What is relevant to our work is that all newly activated cytotoxic T lymphocytes (CTLs) exit the corresponding lymph nodes into lymph via lymphatic ducts before they enter circulation via the great veins, and then flow through the pulmonary circulation (Figure 1). Under resting non-inflamed conditions, re-circulation of lymphocytes between lungs and blood is very rapid, with the average residence time in the lungs less than *one minute* (16).

After leaving the heart and lungs, the traveling CTLs continue to flow into systemic circulation, followed by their ultimate but not instantaneous homing in the corresponding infectious or tumor sites. Indeed, lymphocytes on average must pass via vasculature of the lung or liver about 10 times or even more times (15) before they migrate to one of the secondary lymphoid tissues (12)[BOX 14.2]. For example, it was shown that if anti-tumor CTLs were activated in the breast, they would perform on average about eight circulatory transient cycles before extravasation into the tumor site (15).

Before reconciling the experimental observation (O1) with these studies, we have to briefly discuss a unique role that the lung plays in the physiology and immunology of trafficking lymphocytes under both resting and inflamed conditions.

Experimental studies have revealed that different subsets of lymphocytes, including naïve/memory/activated effector T cells, transiently accumulate in the lungs (17, 18) both by means of and, what is also extremely important, without specific antigen-dependent recruitment of CTLs to the lung (19). Anderson et al. (19) further discuss “numerous observations indicating that T cell trafficking withing the lung is starkly different from what is known about T cell trafficking in most nonlymphid tissues,” including the fact that lymphocyte extravasation into the lung is chemokine independent (20, 21). So, one must revisit the observation (O1) by taking into account the unique role that the lung may play in lymphocyte retention even in the absence of influenza A related antigen-induced chemokine gradients that would additionally force anti-melanoma CTLs to extravasate into the lung epithelium, should influenza A infection be present.

Unfortunately, the above results and the unique role of the lung to transiently retain lymphocytes still do not explain the difference in the observations (O1) and (O2), nor they explain the observation (O3), for the following reasons.

First, concerning the observations (O1) and (O2), both anti-melanoma and anti-infection CTLs should follow the same pattern of multiple vascular re-circulation cycles as discussed above (Figure 1). However, under similar re-circulation patterns, the presence of IAV infection impedes tumor clearance, while, at the same time, both IAV and another concomitant infection (e.g., VACV) are cleared efficiently as one infection would be cleared in the absence of another. Specifically, the question “Why are anti-melanoma CTLs retained in the infected lung, while anti-VACV infection CTLs are not?” remains unanswered.

Given the large literature body on the importance of PD-1 receptors in immune response and the observations (O3) and (O5), we decided to explore theoretically whether molecular signaling pathways initiated by PD-1 ligated with PD-L1 would provide at least one plausible mechanism to explain the results obtained in Kohlhapp et al. (1).

We have excluded PD-L2 from our model and only consider PD-L1 in the analysis that follows. Indeed, PD-L2 has restricted expression on macrophages, dendritic cells (DCs), and mast cells, while PD-L1 is expressed more broadly in order to mediate T cell tolerance in non-lymphoid tissues (12, 22). Besides, mathematical simulations based on the biophysical and expression data have revealed an unexpectedly limited contribution of PD-L2 to PD-1 ligation during interactions of activated T-cells with APCs (23).

To this end, the immune system has apparently evolved the inhibitory PD-1/PD-L1 pathway as a result of the need to control the degree of inflammation at locations expressing the antigen in order to secure normal tissue from damage and also to maintain peripheral tolerance (4, 22). This includes the constitutive expression of PD-L1 in large quantities in various tissues such as lungs, pancreatic islets, ovary, colon, etc. (24–29) by which cross-reactive effectors that survive positive selection are also muted to maintain the peripheral tolerance (2).

### 2.3. Incoherent Feed-Forward Regulation of PD-1 Expression

**Figure 2:**
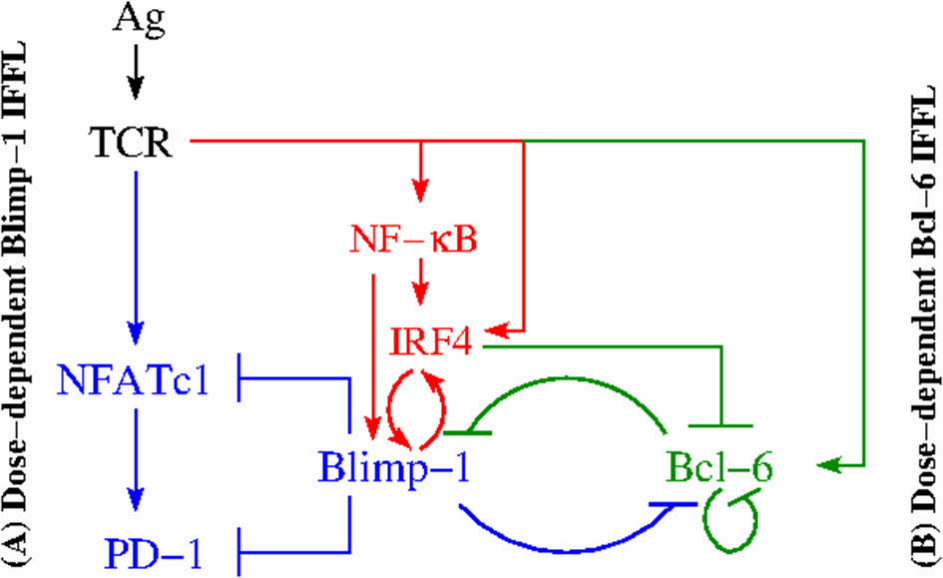
Regulation of PD-1 expression. Two different IFFLs, sharing a common set of species and regulatory activities highlighted in red, are presented. Both IFFLs are activated by the same input (Ag). The left hand side **(A)** depicts a dose-dependent biphasic activation of PD-1. The elements of the corresponding IFFL are highlighted in blue and red colors. When the input, the Ag dose, increases, the output, the PD-1 level, first also increases but then subsequently decreases. The right hand side **(B)** corresponds to a dose-dependent activation of Bcl-6. The elements of the corresponding IFFL are highlighted in green and red colors. Over a certain range of input dose, the Ag level, the output, in this case Bcl-6 level, increases but with a subsequent increase in the Ag dose, the Bcl-6 level then decreases.

PD-1 expression on CD8+ T cells is known to be regulated at the level of transcription of its gene *pdcd1* (30). More precisely, two upstream conserved regulatory CR-B and CR-C regions (30) are key for PD-1 expression in response to CD8+ T cell activation. Specifically, TCR signaling induces PD-1 gene expression through the transcriptional activator, Nuclear Factor of Activated T cells, cytoplasmic 1 (NFATc1) (Figure 2), which binds to CR-C after translocation to the nucleus (30, 31).

Next, the down-regulation of PD-1 during acute infection (32) suggests that there exists a mechanism that directly represses its expression after initial activation events. Indeed, Blimp-1 (B Lymphocyte-Induced Maturation Protein 1) (33) has been found to be induced during the *later* stages of CD8+ T cell activation and was shown to be required for the efficient terminal differentiation of effector CD8+ T cells (30). When Blimp-1 is suppressed, the same data suggest that in the absence of Blimp-1, PD-1 expression is maintained by NFATc1 (Figure 2).

For the sake of completeness, we recall that the existing data also suggest that Blimp-1 represses PD-1 gene expression in CD8+ T cells using three distinct molecular mechanisms (30):

(1) regulation of the expression of PD-1's activator NFATc1;
(2) alteration of the local chromatin structure; and
(3) eviction of the activator NFATc1 from its site that controls PD-1 expression.

In addition, Blimp-1 has been found to be a transcriptional antagonist of proto-oncogene Bcl-6 (B cell lymphoma 6 transcription factor), and vice versa (Figure 2) (i.e., Blimp-1 and Bcl-6 are known to mutually repress one another) (34–38). Specifically, Blimp-1 can bind to the *bcl6* promoter (39).

Although it is not known exactly how Bcl-6 inhibits Blimp-1 in T cells, it is well known that in B cells Bcl-6, in association with a corepressor MTA3, represses *prdm1* by binding to sites in *prdm1* intron 5 and intron 3 (34, 40, 41). We take this fact into consideration because signaling pathways and their activation are similar in both B and T cells (12). Additionally, Bcl-6 binds its own promoter and inhibits its own transcription (Figure 2), thus implementing an autoregulatory loop (42, 43) (Figure 2).

Competing with Bcl-6 for intron 5 in *prdm1*, IRF4 (Interferon Regulatory Factor) (44–47) is shown to be a direct activator of *prdm1* (Figure 2) by binding to a site in intron 5 (34). At the same time, IRF4 directly represses gene *bcl-6* by binding to a site within its promoter (34, 45), which is rich in IRF4-binding sites (43).

Because IRF4 is known to be activated both directly via TCR and by NF-κB (48, 49), we have then sought to determine who activates NF-κB in this context and found that NF-κB is activated by TCR signaling (34, 37, 50, 51). Several potential NF-κB binding sites in the *prdm1* promoter have been suggested. It is also known that IRF4 can bind to its own promoter, supporting a positive feedback mechanism by which high IRF4 expression can be maintained (43, 52).

There are additional signaling routes leading to the activation of IRF4 (e.g., via Akt-mediated pathways) which are not discussed here (34).

After a careful analysis of the reconstructed molecular interactions, we have come to the conclusion that this intricate reaction network consists of two subnetworks (Figure 2). Both subnetworks have the same input from the activated TCR, while the outputs of the subnetworks are different. Namely, PD-1 is the output of the subnetwork color-coded in blue and red, while Bcl-6 is the output of the subnetwork color-coded in green and red. The two subnetworks share a number of common species and interact with one another via repressive interactions mediated by the three key species color-coded in red, (i) IRF4, (ii) Blimp-1, and (iii) Bcl-6.

Each of the two subnetworks corresponds to a gene-regulatory network (GRN) motif known as an incoherent feed-forward loop (IFFL) (6). Because the PD-1 circuit is formed of two such IFFLs, we call it a Double Incoherent Feed-Forward Loop (DIFFL).

Our IFFL network may be viewed as a mechanistic instantiation of a conceptual signal discrimination model based on a competition between “excitation” and “de-excitation” factors possessing different response kinetics, as initially introduced by Grossman and Paul (3). The latter concept has been gradually applied successfully in multiple studies since 1992 as reviewed in Grossman and Paul (5). In that sense, we address with our model the following goal formulated in Grossman and Paul (3): “More explicit rules of organization, or models, need to be explored. Such rules should suggest, in particular, how the functional segregation of immunological responses may reasonably come about.”

### 2.4. PD-1 Expression Within Different Inflammatory Contexts

**Figure 3:**
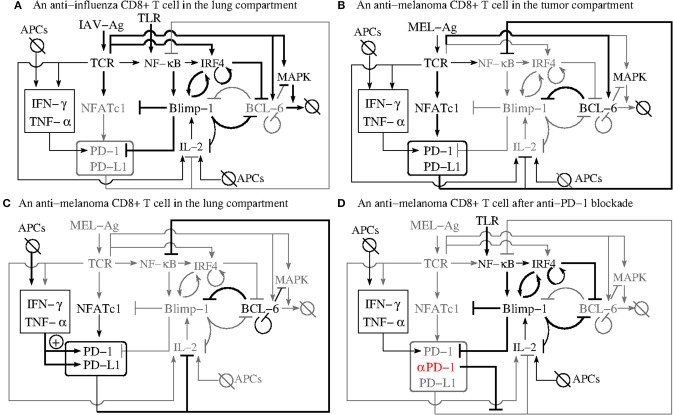
The PD-1 DIFFL motif in the context of complex influenza-tumor interactions. **(A)** Shows the PD-1 DIFFL response in an anti-influenza CD8+ T cell in the infected lung. **(B)** Shows the response of the PD-1 DIFFL circuit in an anti-tumor CD8+ T cell in the TME. **(C)** Shows the PD-1 DIFFL response in an anti-tumor CD8+ T cell in the influenza-infected lung. **(D)** Shows the PD-1 DIFFL response in an anti-tumor CD8+ T cell in the influenza-infected lung after PD-1 blockade. Gray color corresponds to weak or disabled reactions shaped by the given inflammation context. Symbol + inside a circle in **(C)** shows the additional PD-1 activation route initiated by external cytokines in the case when the Blimp-1 mediated repression of PD-1 expression is absent. This route does not play any significant role in the case when the expression of PD-1 is suppressed by active Blimp-1 as in **(A)**. Arrows denote activation, and barred lines denote repression. The abbreviation APCs stands for (influenza) Antigen Presenting Cells.

We next attempt to validate the PD-1 DIFFL motif (Figure 2) against all observations reported in Kohlhapp et al. (1) by following the falsification and validation methodology (53), which is also fundamental to any modeling study. Figure 3 will be instrumental in our analysis that follows.

Figure 3A shows a biochemical reaction network reconstruction customized for the case of an anti-influenza cytotoxic effector T cell, T_EFF_, in the presence of large amounts of cognate Ag in the infected lung. In this case, the immunological complexity of interactions involving cytokines is already overwhelming (5, 54–58). For example, IL-2 activates and is simultaneously repressed by active Blimp-1 both directly and indirectly (31, 59).

The abundance of the cognate viral Ag in the infected lung leads to a strong TCR activation which, in turn, results in the simultaneous activation of Blimp-1 and degradation of Bcl-6 (section 2.3) followed by suppression of PD-1 transcription with its subsequent degradation. The biochemical detail can explains transient and rapid PD-1 expression followed by downregulation of PD-1 expression in the presence of acute infection (32), see also section SI-1.2.

All this may also explain why anti-infection CD8+ T cells are not exhausted during the first phase of the biphasic response of the PD-1 DIFFL-circuit (section 2.3) despite the fact that bystander and tissue cells express large amounts of PD-L1 caused by large concentrations of pro-inflammatory cytokines such as INFγ (SI-1.1). Recall that large amounts of PD-L1 are already constitutively expressed in the lung under resting condition in the absence of any infection (section 2.3).

Figure 3B shows the response of the reconstructed circuit in the tumor microenvironment (TME). Specifically, anti-melanoma CD8+ T cells overexpress PD-1 in the presence of large amounts of tumor-specific cytokines such as IL-6, a well-described regulator of Bcl-6 expression (38). Due to relatively low levels of tumor Ag and a weak self-Ag TCR signal (60) of anti-tumor CD8+ T cells, the TCR is not activated strongly enough to activate Blimp-1 and, at the same time, the weak activation of the TCR sets the first phase of the biphasic response of the dose-dependent PD-1 DIFFL motif (Figure 2).

Indeed, the PD-1 DIFFL strongly activates Bcl-6 for small and medium TCR strengths, and weakly activates Bcl-6 for high activity levels of TCR. As a result, Bcl-6 is overexpressed, while Blimp-1 is not expressed in the melanoma TME (38), which leads to the overexpression of PD-1 on the surface of anti-tumor CD8+ T cells.

Figure 3C shows the PD-1 DIFFL in an anti-melanoma T_EFF_ relocated into the infected lung. In this case, the conditions discussed just above to introduce Figure 3B play the role of a spark plug that activates the transcription of Bcl-6, which represses *prdm1* even after the relocation of the anti-tumor T_EFF_ into the lung.

These relocated T_EFF_ can now sense the elevated levels of INF-γ and TNF-α, which are abundant in the infected site, and which are produced by professional antigen presenting cells (APCs) (section 2.2).

The cytokines strongly stimulate the expression of both PD-1 and PD-L1 (61), as well as maintain the expression of PD-1 on the surface of the anti-melanoma T_EFF_, initially sparked by the ligation of TCRs with cognate tumor Ags during the time when the T_EFF_ cells were present in the TME before their relocation to the lung.

Because the tumor Ag is absent from the infected lung, the TCR is not ligated, and, hence, all routes leading to the activation of Blimp-1 and IRF4 are disabled. We can thus propose that the major route contributing to PD-1 overexpression here is mediated by INFγ and TNFα. The corresponding PD-1 expression activation route is marked by sign + inside a circle in Figure 3C.

Recall that large quantities of PD-L1 are constitutively expressed in the lung already under resting conditions (section 2.2). PD-1 mediated control of immune responses depends on interactions between PD-1 on CD8+ T cells and PD-L1 in tissues (62). Importantly, such PD-1:PD-L1 interactions can result in CD8+ T cell motility paralysis (8, 28, 63).

**Figure 4:**
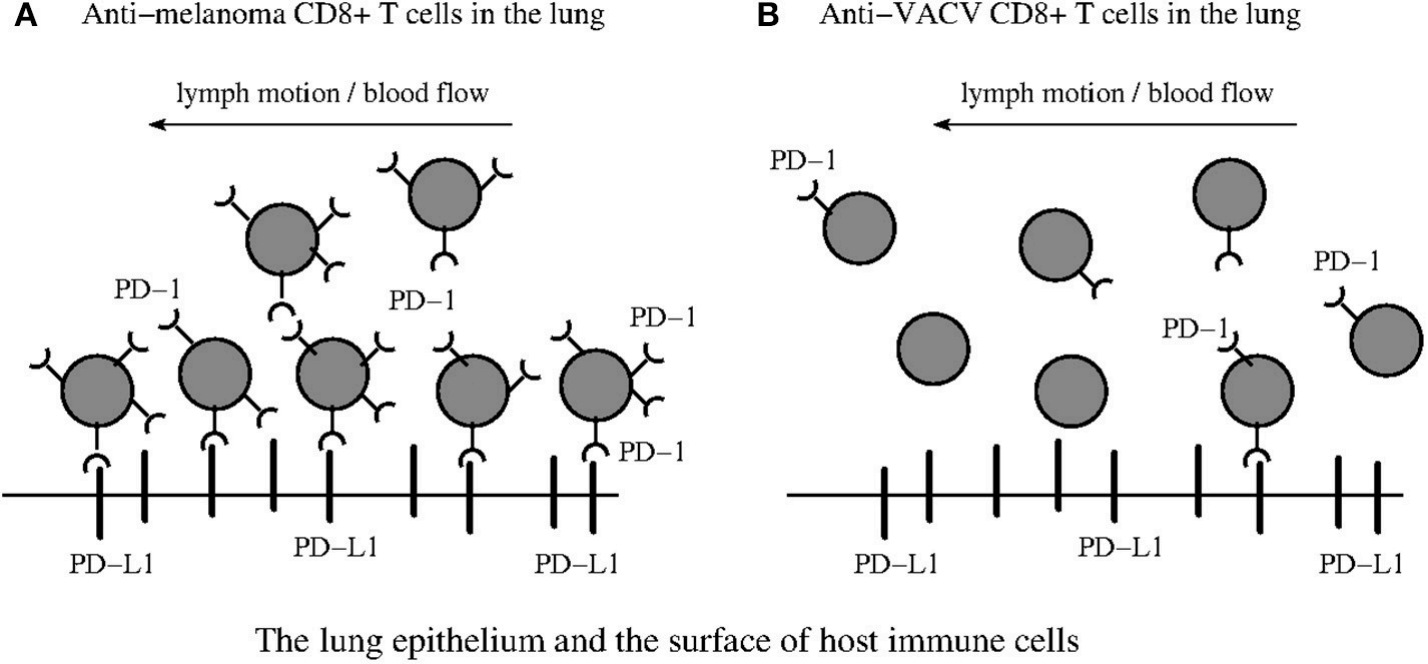
PD-1:PD-L1 induced paralysis of the anti-tumor exhausted CD8+ T cells in the infected site. **(A)** Suggests that anti-melanoma T_EFF_ cells become paralyzed in the infected lung. In contrast, **(B)** suggests that anti-VACV T_EFF_ studied in Kohlhapp et al. (1) can freely enter and leave the infected lung with the lymph motion and blood flow due to the lack of large amounts of PD-1 receptors on their surface. The immune suppressive environment (4) induced by inflammation in the infected lung is caused by multiple interactions between PD-1 receptors, expressed in large quantities on the surface of the anti-melanoma T_EFF_, and the PD-L1 ligands expressed in large quantities on the surface of various host immune cells (macrophages, DCs, and MDSCs) and the epithelium (29).

We introduce the paralysis mechanism (Figure 4) in detail in (O1-M6) (section SI-1.3) and believe that this mechanism can provide a valuable insight into the previously unrecognized factor contributing to the retention of anti-melanoma CD8+ T cells shunted to the influenza A infected lung (1). Of course, other yet unknown mechanisms may exist and need to be elucidated in order to provide a more complete explanation of the retention effect (1). Therefore, additional experimental observations should be obtained.

The study conducted by Cheng et al. (23) reports that “it now seems that very stable complexes are not prerequisite for potent inhibitory PD-1:PD-L1 signaling” because measurements of the human and mouse PD-1 binding to PD- L1 affinities suggest that potent inhibitory signaling can be mediated by weak interactions.

Zinselmeyer et al. (8) further stress: “Prolonged motility arrest is an excellent host strategy to decrease T cell efficiency and likely facilitates exposure to multiple regulatory pathways. PD-1:PD-L1 blockade is known to restore function to virus-specific and tumor-specific T cells, and has shown some promise in recent clinical trials.”

Although dissociation and association of the complex PD-1:PD-L1 are assumed to be fast (64, 65), this however does not preclude the long-known loss of T cell motility due to multiple PD-1:PD-L1 interactions (66, 67).

Figure 3D shows the PD-1 DIFFL in an anti-melanoma T_EFF_ cell in the infected lung after administration of PD-1 (αPD-1) blockade. Recall that the NF-κB pathway is downregulated in exhausted CD8+ T cells (38). To this end, the PD-1 blockade (marked by symbol αPD-1 color-coded in red) in Figure 3D, removes the brake (68) from the corresponding T cell signaling pathways (see section 2.1, observation O(3), and Table SI-1.1) leading to overexpression of NF-κB (66, 69). Additionally, NF-κB activation is positively regulated through TNFR (TNF Receptor) and TLR (Toll-like Receptor) sensing TNFα and viral materials in the infected lung, respectively (70–72).

As discussed earlier, NF-κB activates IRF4 (34), and the latter directly represses Bcl-6 (34). In turn, the repression of Bcl-6 removes the brake from the overexpression of Blimp-1, which then leads to reduced numbers of PD-1 receptors on the surface of reactivated anti-melanoma effector cells. This may allow the reactivated T_EFF_ to become mobile (Table SI-1.1) with a potential to relocate back to the melanoma TME with the lymph flow and blood circulation as discussed in the mechanism (O1-M6). Indeed, it is well known that after the T_EFF_ re-circulation in the blood (15), effector T cells are preferentially found in the lymph nodes in which their activation occurred, and in the area drained by those lymph nodes (73).

The above conclusions are also based on the experimental evidence that PD-1:PD-L1 interactions contribute to reduced T cell motility on day 7, and therapeutic blockade of PD-1:PD-L1 restore CD8+ T cell motility within 30 min (8). Although we use the references (8, 63) in order to support our hypotheses, additional experimental research is needed to understand deeper the paralysis phenomenon (28, 63).

We conclude our discussion of the PD1 DFFIL motif by noting that the core of the reconstruction (Figure 2) fits well to all discussed inflammatory contexts (Figure 3).

### 2.5. Probing Immunobiochemical Reconstruction Modeling

Our modeling goal here is quite simple. Given the discussed specificity of PD-1 expression (section 2.4) with respect to different amounts of antigen available in the medium and different values of TCR affinity in terms of the values of the off-rate constant *k*_off_ for the Ag:TCR bond (74, 75), we focus on the analysis of the dependence of the levels of key species, Bcl-6, IRF4, Blimp-1, and PD-1, on the two parameters, (i) the antigen concentration, Ag, and (ii) the values of *k*_off_ defined in sections SI-2 and SI-3.

#### 2.5.1. Modeling PD-1 Expression in the Absence of PD-L1

We first consider the case when the PD-1:PD-L1 interaction is absent from the model by setting ϕ_*L*_(*P*)≡Φ(*P*)≡1 corresponding to the condition *L* = [PD-1:PD-L1] = 0 in both Equations (SI-2.1c) and (SI-2.2a).

**Figure 5:**
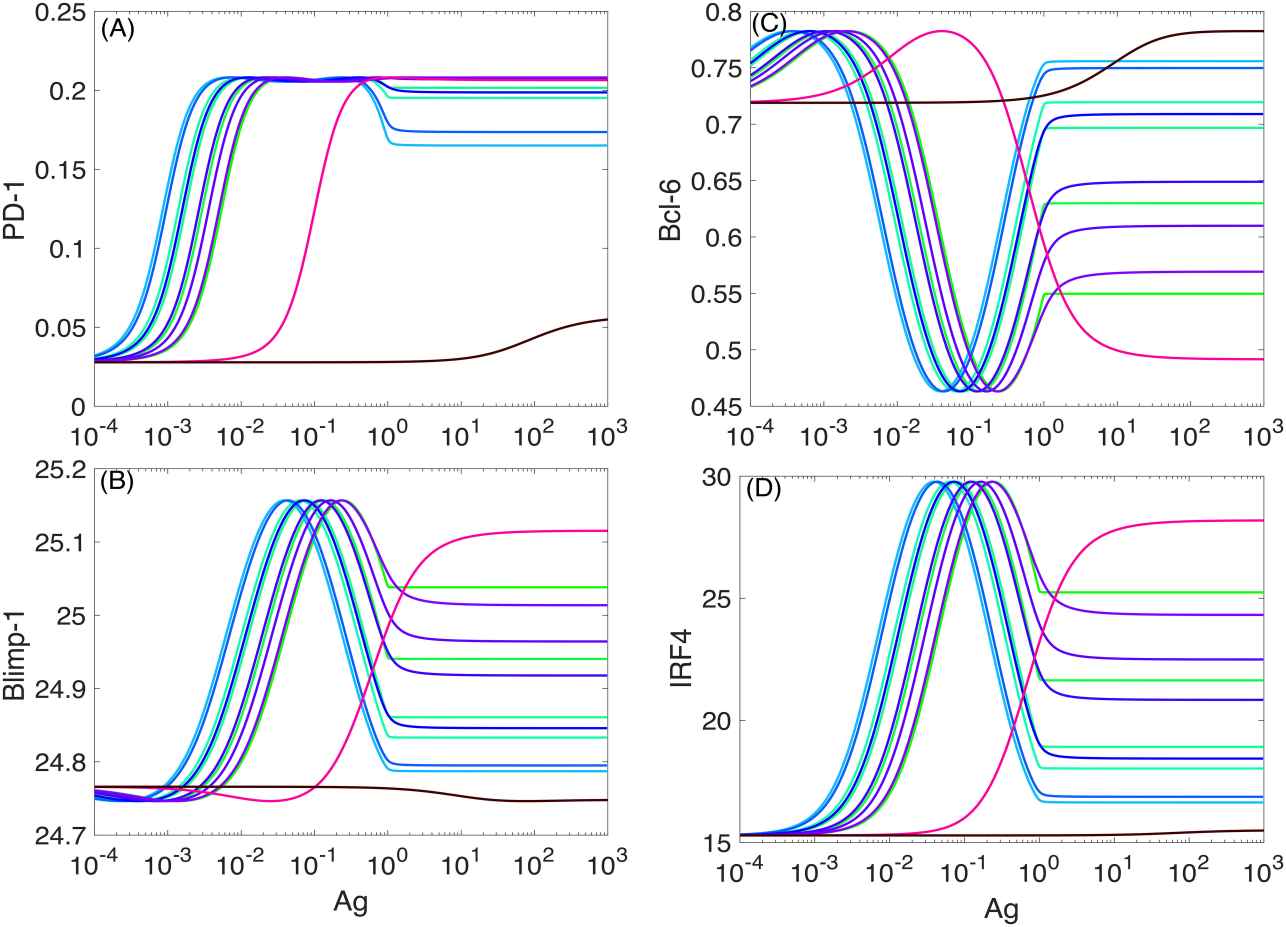
PD-1 DIFFL responses in the absence of PD-1:PD-L1 interaction. The color-coded plots corresponds to the PD-1 DIFFL-induced adaptation with respect to increasing Ag-levels. To obtain a full adaptation, approximately a 10^3^-fold increase in the Ag-level is required. Four different (bottom-up) shades of green color correspond to $k_{\text{off}}=10^{-4},2.03\times 10^{-4},4.13\times 10^{-4},$ and 5.88 × 10^−4^, respectively. Two shades of blue color correspond to $k_{\text{off}}=2.43\times 10^{-3}$ and 7.01 × 10^−3^, respectively. Four (top-down) shades of purple color correspond to $k_{\text{off}}=2.03\times 10^{-2},4.13\times 10^{-2},5.88\times 10^{-2}$, and 8.38 × 10^−2^. Magenta color corresponds to *k*_off_ = 1.0. Black color corresponds to *k*_off_ = 49.24. **(A–D)** Correspond to the levels of four species, PD-1, Blimp-1, Bcl-6, and IRF4, computed from the model developed in SI2, respectively.

Typical plots for the (non-dimensional) steady-state (76) concentration levels of PD-1, Bcl-6, Blimp-1, and IRF4 in the absence of the PD-1:PD-L1 interaction and at the different values of *k*_off_ are shown in Figure 5. The model's nondimensionalization is done in sections SI-2 and SI-3.

We next discuss the case of small values of *k*_off_ from the set of the values given in the legend of Figure 5. We observe from Figure 5 that the level of PD-1 (Figure 5A) becomes rapidly elevated already at very small values of the scaled Ag-concentration (section SI-1). A further increase in the scaled Ag-concentration results in the formation of the PD-1 level plateau, followed by a drop in PD-1 levels.

The increase in the level of PD-1 (Figure 5A) is fully aborted when the level of Blimp-1 (Figure 5C) reaches the threshold sufficient to suppress PD-1 expression initiated by TCR activation. We interpret the top (left) plateau in the level of PD-1 (Figure 5A) as corresponding to the homeostasis maintained by both the PD-1 DIFFL and the negative feedback activation of TCR which we discuss shortly below. At the same time the bottom (right) plateau in the level of PD-1 (Figure 5A) can be interpreted as an adaptation to high levels of Ag (3), a direct consequence of adaptive properties of IFFLs (6, 77–82).

We further observe that in complete agreement with the theory of IFFLs demonstrating biphasic steady-state behavior (6, 77, 78), the levels of Blimp-1 and IRF4 first increase and then decrease, and, at the same time, the level of Bcl-6 first decreases and then increases, while the level of Ag is constantly increased. Remarkably, the levels of all the three species almost perfectly adapt to their respective original states formed initially at very low levels of Ag, when the level of Ag becomes high enough to establish adaptation. A similar adaptive phenotype is discussed using an example of a generalized enzyme network in Chiang et al. (79).

Consider now the case of large values of *k*_off_ from the set of the values given in the legend of Figure 5. In this case, the response of the PD-1 DIFFL becomes abnormal, when all remarkable adaptive properties are completely lost. Even in the case of a very large value of *k*_off_, the model predicts a tonic expression of PD-1 corresponding to very small nonzero values coded in black color in Figure 5A. We believe that this tonic expression of small PD-1 levels can be attributed to the immune tolerance discussed in section SI-1.

**Figure 6:**
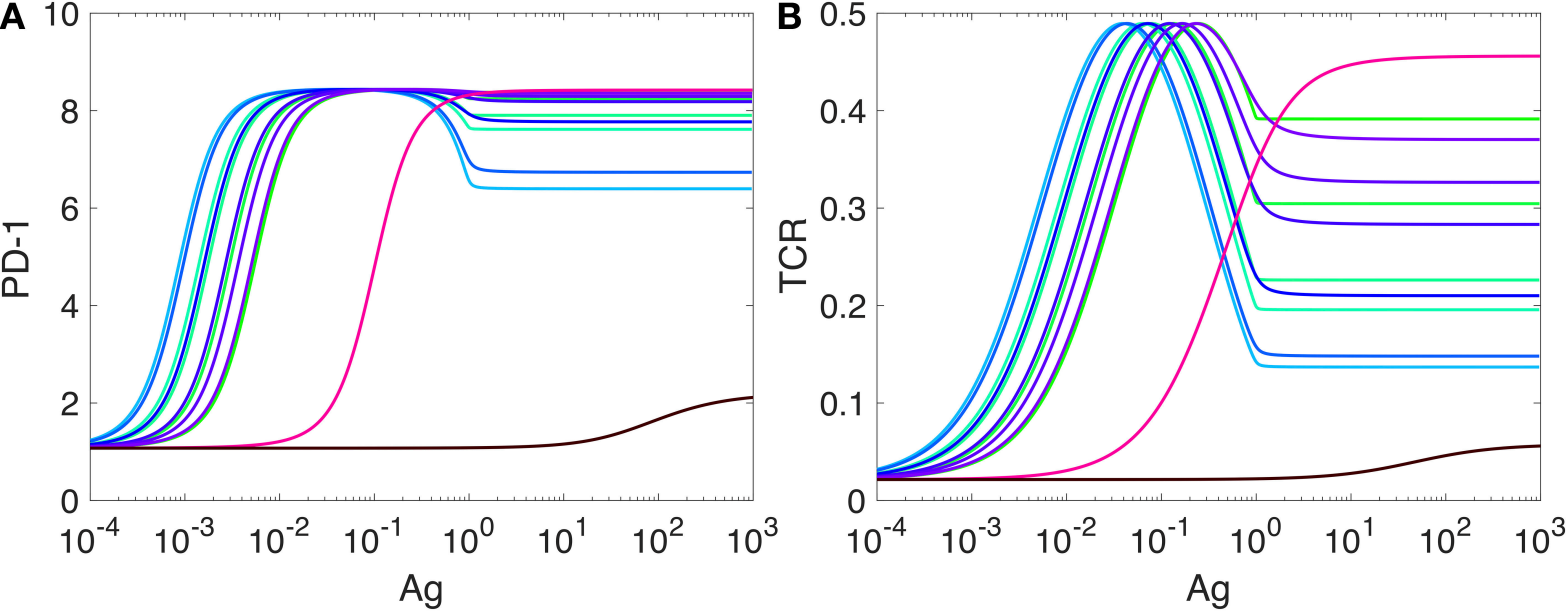
Expression of PD-1 in the case when the expression of IRF4 is disabled. The levlel of PD-1 receptors in **(A)** is computed from our model developed in SI2. The level of TCR activity in **(B)** is computed from the model developed in Lever et al. (74) as explained in SI-2. All other explanations and parameter values are as in Figure 5.

To better see the role of IFR-4 and its impact on the level of PD-1, we then completely disabled IRF4 by setting the value of the parameter *k*_*b*_ to zero, *k*_*b*_ = 0 in the Equation (SI-3.1d). This computational experiment can be thought of as an “*in silico* IRF4-knockout.” The corresponding plot of PD-1 levels against the Ag-concentration is shown in Figure 6A.

Surprisingly, the shapes of all PD-1 level plots obtained for the same set of *k*_off_ values as in Figure 5 are preserved, and only the magnitudes of the corresponding levels are changed by a factor of 40 or more.

Motivated by these computational predictions, we checked if IRF4 knockout results were previously reported in the literature and found that *irf4*-deficient CD4+ T cells display increased expression of PD-1 associated with T cell dysfunction (83, 84). However, the role of IRF4 is still poorly understood as it can be completely opposite in the cases of acute and chronic infections (83, 85).

The second interesting observation (Figure 6B) is that while the PD-1 DIFFL regulatory function is lost due to *in silico* knockout of IRF4, the adaptation of PD-1 expression with respect to Ag levels (Figure 6A) is still preserved by the negative feedback regulation of TCR activity (Figure 6B) (5, 86–88). Both the TCR activation and the negative feedback are interpreted as another IFFL in Lever et al. (74). Collectively, we can thus conclude that the PD-1 transcription and its adaptation to high levels of antigen is regulated by multiple incoherent feed-forward loops.

#### 2.5.2. Modeling PD-1 Expression in the Presence of PD-L1

**Figure 7:**
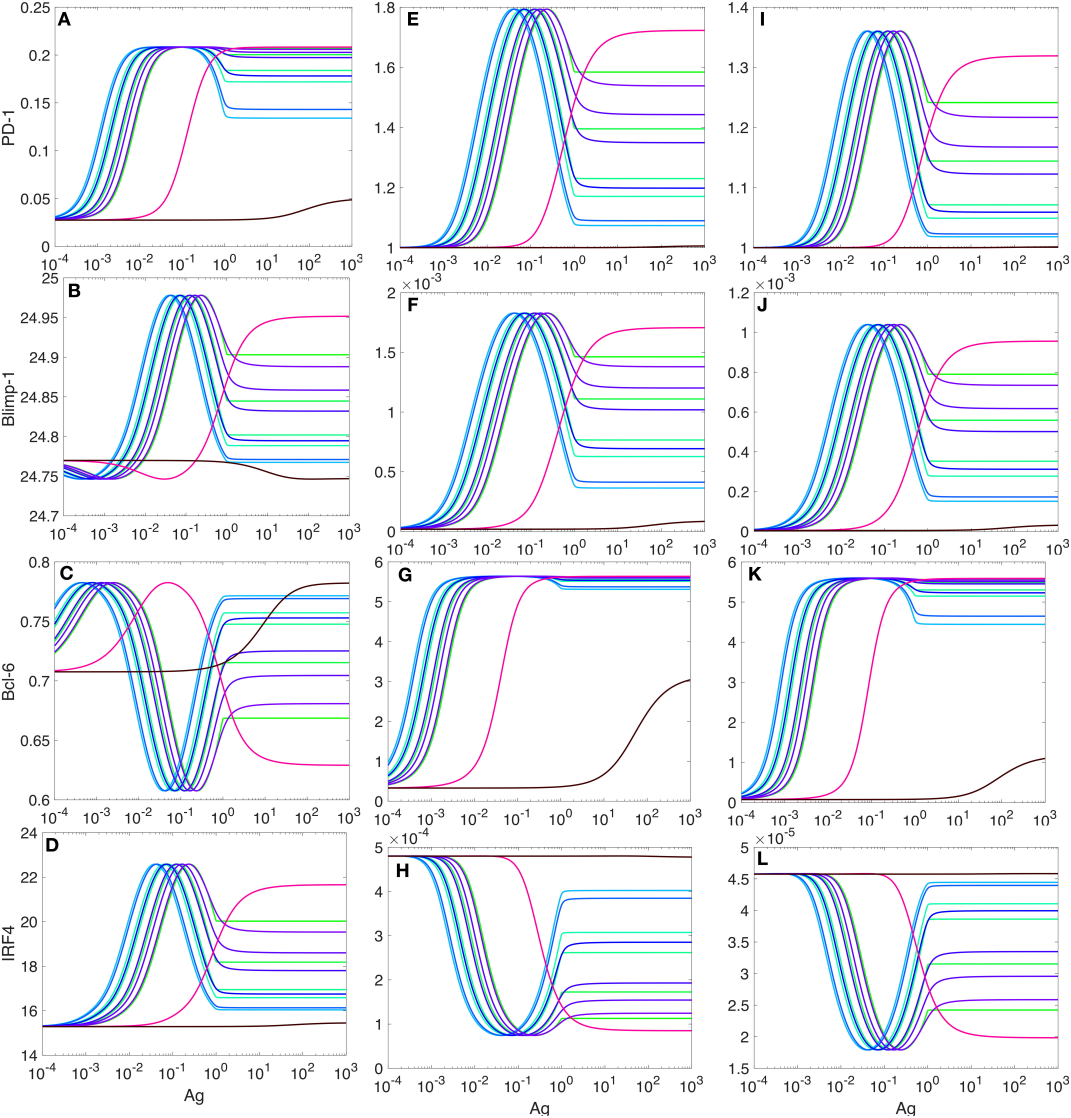
PD-1 DIFFL responses in the presence of PD-1:PD-L1 interaction. **(A–D)** Correspond the case when 20% of PD-1 receptors are ligated with PD-L1. **(E–H)** Correspond the case when 50% of PD-1 receptors are ligated with PD-L1. **(I–L)** correspond the case when 90% of PD-1 receptors are ligated with PD-L1. All other explanations are provided in the legend for Figure 5.

We observe that in the presence of PD-1:PD-L1 interactions, the maximum levels of PD-1 and Bcl-6 increase (by a factor of 6.75 and 7.86, respectively, but, of course, these numbers are only meaningful in our model and with the parameters used, and they do not have biological significance) (Figure 7). At the same time, the levels for Blimp-1 and IRF4 are negligibly small, which allows us to interpret that the transcription of these two species is almost fully suppressed (Figure 7).

From our comparison of the PD-1 level plots in Figures 5, 7, we can conclude that the PD-1:PD-L1 interaction plays the role of an amplifier of transient activation of PD-1 transcription, initiated by the ligation of TCR with Ag presented with an MHC (section SI-1).

PD-1:PD-L1 interactions may terminate signal transduction pathways, including those pathways that lead to the activation of IRF4 and Blimp-1, by recruiting phosphatases (68, 89, 90).

Our last computational experiment compares quantitatively the PD-1 level on the surface of an anti-melanoma CD8+ T cell shunted to the lung with the PD-1 level on the surface of an anti-influenza CD8+ T cell in the lung under the same conditions.

To conduct the computational experiment, the following conditions were taken into consideration: (i) the absence of distant tumor Ag in the lung, leading to the shutting down of the TCR signal (*U* = 0 in the Equations (SI-2.1a–d), (ii) the abundance of inflammatory cytokines, including TNFα and IFNγ, known to induce the expression of both PD-1 and PD-L1 (SI-1), and (iii) the abundance of IL-2, which induces Blimp-1 (SI-1).

To account for the abundance of the lumped TNFα and IFNγ species, we have replaced the rate constant σ_*p*_ in the Equation (SI-2.1b) by the rate expression (SI-2.6). To account for the abundance of IL-2 in the lung compartment, we have increased the value of the parameter *a*_*b*_ by a factor γ in Equation (SI-2.1d). In this case, we assumed that IL-2 was secreted by activated T cells (50) and, hence, IL-2 affected Blimp-1 expression through autocrine and paracrine signaling, depending on the TCR activation strength.

In the case when the value of the parameter γ was set to one, the level of PD-1 was increased by a factor of 6 compared with the maximum level of PD-1 shown in Figure 7 for both anti-influenza and anti-melanoma cases. So, we can conclude that just the PD-1 DIFFL alone is not enough to counteract the effect of the pro-inflammatory cytokines. Only when a “strong action of IL-2” was taken into consideration by setting γ>5, 000, the level of PD-1 was suppressed for anti-influenza T cells.

## 3. Discussion

Below we discuss our modeling studies conducted in order to complement our immunobiochemical reconstruction toward a better understanding of the previously unrecognized acute non-oncogenic infection factor (1). We then discuss potential implications of our research to further stimulate ongoing efforts toward developing and improving physiological and functional cure approaches based on the host's ability to eliminate non-self foreign invaders and, at the same time, the host's inability to install strong altered-self (cancer) responses (2).

### 3.1. What We Learn From the Model

**Figure 8:**
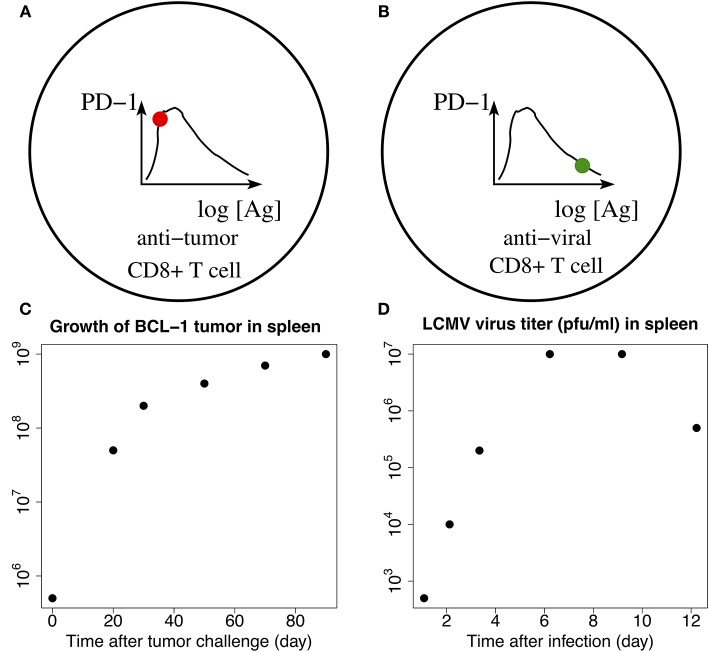
Schematic illustration of the adaption loss/gain hypothesis. Solid filled circles on the corresponding graphs of PD-1 receptor levels (top panels), plotted vs. the log-concentrations of Ag, correspond to the levels of PD-1 receptors on anti-melanoma **(A)** and anti-virus **(B)** CD8+ T cells, respectively (top panels). Phenotype **(B)** corresponds to a fully developed adaptation of the PD-1 expression with respect to the increasing levels of Ag, while phenotype **(A)** is characterized by the lack of such adaptation. Bottom **(C,D)** show time-dependent levels of BCL-1 tumor cells (left) and LCMV virus titers (right) in the spleen. The data points are digitized from the corresponding plots in Kuznetsov et al. (91) and Bocharov et al. (92), respectively. Comparing **(C,D)**, we observe that the changes in the tumor Ag levels within the first 7 days are small, corresponding to the fold change less than 10 as seen from **(C)**. At the same time, the viral Ag levels change significantly, corresponding to the 10^4^-fold increase during the first seven days as seen from **(D)**. The small 7-day tumor Ag-level increase shown in **(C)** corresponds to the red solid “snapshot” circle in **(A)**, while the large 7-day increase in the viral Ag level shown in **(D)** corresponds to the green solid “snapshot” circle in **(B)**. Additional detailed explanations of **(A–D)** are provided in the main text.

Our PD-1 DIFFL reconstruction (Figure 2), when combined with the mathematical modeling (Figures 5, 7), suggests that it is the loss of Ag dose-dependent adaptation of the expression of PD-1 receptors in the anti-tumor CD8+ T cells that could be one of major factors resulting in the multiple effects in the presence of acute non-oncogenic infection (1). Specifically, in the case of acute infection, the level of PD-1 receptors on the surface of Ag-experienced anti-infection CD8+ T cells first increases and then decreases to lower levels in the course of the virus replication (Figure 8B), the hallmark of a fundamental biological adaptation (3). Therefore, based on the discussion around Figure 3, we can conclude that chances that the cells with the phenotype shown in Figure 8B will loose their motility due to PD-1:PD-L1 interactions in the infected lung are low (Figure 4).

In contrast, in the case of Ag-experienced anti-tumor CD8+ T cells, due to the much smaller levels of tumor antigens presented with MHCs in the TME, the strength of the TCR signal in anti-tumor CD8+ T cells may not be enough to activate Blimp-1 and IRF4 species to suppress PD-1 expression (Figures 2, 3). The lack of the expression of Blimp-1 in melanoma is known experimentally (38). As a result, chances that T cells bearing large numbers of PD-1 receptors (Figure 8A) will be paralyzed in the infected lung due to PD-1:PD-L1 interactions are high.

Importantly, the higher levels of PD-1 receptors on anti-melanoma CD8+ T cells compared with much lesser levels of PD-1 receptors on anti-influenza CD8+ T cells co-localized in the same infected lung were observed in Kohlhapp et al. (1). This supports the two different phenotypes shown in Figures 8A,B, respectively.

Our quantitative estimates obtained from the model (Figures 5, 7) show that the Ag level should be increased by several orders of magnitude required to move the Ag-experienced T cell from phenotype (A) to phenotype (B) (Figure 5). This means that at least a 1000-fold increase in cognate Ag levels (Figures 5, 7) may be required for the adaptation of PD-1 expression to strong antigen-mediated stimulation.

Although more research into the novel adaptation effect illuminated by our model as well as into the lymph motion (93, 94) and molecular mechanisms by which cells are rapidly moved with the blood (95) is undoubtedly needed, we believe that it is worth providing some “biological” numbers that support our findings. For example, for the LCMV system, a gold standard for infectious biology, the virus titer was increased by factor about 10^3^ from day 2 to day 5 (92, Figure 4.4). We digitized the corresponding data points and plotted them in Figure 8 next to Figure 8B. Similar data are reported for influenza A infection (96, 97). The examples of the population measurements are well translated to our modeling studies because in all cases we use dimensionless ratios of the corresponding concentrations.

Of course, one also needs to make sure whether a T cell would be capable to provide a large number of TCRs sufficient to accommodate the above huge increase in Ag-levels. Indeed, the typical number of TCR molecules is estimated in the range of 3 × 10^4^ (98), which is a reasonable number to match up with the model-suggested transition from phenotype (A) to phenotype (B) shown in Figure 8. At the same time it is highly unlikely for tumor cells to divide as fast as the viruses do to build enough antigen that would be sufficient to change phenotype (A) to phenotype (B) within a few days. Indeed, the doubling time for virus particles can be 43–65 min (99), while the doubling time for malignant mouse melanoma B16 cells may take up to 2.8 days or longer (100, 101).

To support the above argument, we note that (91) uses experimental data where the number of tumor cells is increased by factor about 10^2^ in the time span of just 40 days. We digitized the corresponding data points and plotted them in Figure 8 next to Figure 8A. We can thus conclude that due to our modeling estimations (Figures 5, 7), such a slow increase in Ag levels may not be enough to change between the prototypes shown in Figure 8 for short periods of time (days), when acute infection develops and is cleared (1). Similar data can be learned from other independent studies (102).

Note that the discussed transient elevation of PD-1 receptor levels as function of antigen, Figures 8A,B, was experimentally observed and was also used as a “window of opportunity” in the context of the combined radiotherapy (RT) and anti-PD-1:PD-L1 treatments (103). Our theoretical work provides additional valuable insight into, and add in the development of combined RT/anit-PD-1:PD-L1 therapy.

### 3.2. Harnessing Anti-infection and Anti-bacterial Responses Against Cancer

By addressing the “the previously unrecognized acute non-oncogenic infection factor” revealed through systematically collected heterogeneous experimental data encompassing different pathogens and tumor types (1), we have suggested and discussed concrete molecular mechanisms which allowed us to delineate inherently weak anti-cancer (i.e., altered-self) immune responses from inherently strong anti-infection (i.e., non-self, foreign) responses, including co-infections.

Our findings may thus have potential clinical relevance particularly in the context of ever-expanding immunotherapy efforts and FDA approvals involving PD-1/PD-L1 axis immune checkpoint blockade. Two relevant scenarios to consider, include (1) that patients with cancer treated with such blockade may also be experiencing a concomitant diagnosed or sub-clinical undiagnosed infection in a tissue distant to their tumor, and (2) that selective patients with cancer are being treated with oncolytic viruses (OVs), which preferentially infect tumor cells, but can also infect cells in tissues distant to their tumor (104, 105). In both scenarios, checkpoint blockade may have less-recognized effects discussed here (e.g., releasing the T cell motility paralysis caused by an infection in a tissue distant to the tumor) and thus such blockade may improve patient outcomes, including in the context of combination with OVs (106). As additional clinical information is collected from patients receiving checkpoint blockade (including about infection status and OV viral loads in non-injected sites), future efforts may provide the data necessary to reveal and model this blockade effect further.

We conclude this work with a hope that our theoretic analysis of the newly discovered infection-tumor interaction (1), made by combining solid immunobiochemical reconstruction with appropriate mathematical modeling may also be useful in current developments of both “physiological” and “functional cures” (2). Specifically, our mechanistic molecular-based analysis of the novel immunologic phenomenon uncovers important competing push-pull processes fundamentally inherent in immunity (3–5). We believe that the results reported may have broader implication toward developing (i) *physiological cure* approaches in order to completely eliminate tumors as it happens in the case of rapid (one week long) clearance of acute infection, and, alternatively, toward undertaking (ii) *functional cure* treatments to maintain long-term immunologic control as in the cases of controlled chronic infection and other disorders as, for example, hypertension (7). However, research (1) clearly suggests that all such cures must be developed with care.

## Author Contributions

AZ and ES conceived the work. AZ contributed data. EN and ES designed research and wrote the manuscript. EN analyzed the data and performed the research.

### Conflict of Interest Statement

The authors declare that the research was conducted in the absence of any commercial or financial relationships that could be construed as a potential conflict of interest.
