# Supplementary material for: Immunobiochemical Reconstruction of Influenza Lung Infection—Melanoma Skin Cancer Interactions
Source: Front Immunol. 2019 Jan 28;10:4. doi: 10.3389/fimmu.2019.00004 (PMC6360404; doi:10.3389/fimmu.2019.00004)
Supplement: Supplementary file 1 [file Data_Sheet_1.pdf]

Supplementary Material

Immunobiochemical Reconstruction of Influenza Lung Infection - Melanoma Skin Cancer Interactions

SI-1 A REVIEW OF RELEVANT LITERATURE

SI-1.1 An immunobiochemical reconstruction scope

Given the complexity of the processes relevant to the observations **(O1) - (O5)**, we must limit the scope of our research. Because the interaction between PD-1 and PD-L1 is regarded as a major "T cell brake" (Nirschl and Drake, 2013), and because it is also a central topic in (Kohlhapp et al., 2016), we center our analysis on the construction of the gene regulatory networks (GRNs) directly involved in the expression of PD-1 receptors under different immunologic contexts.

A very important topic that shapes the scope of our work is that T cell activities are controlled at multiple levels. These regulatory controls are necessary to prevent T cells from becoming hyperactivated, causing significant collateral damage to non-target tissue. These types of responses enhance inflammation, resulting in the release of self-antigens from necrotic tissue, increasing the chances for the induction of autoimmune diseases (Liechtenstein et al., 2012). To avoid autoimmunity induced by necrotic tissue, key regulatory T cell inhibitory interactions occur between PD-L1 expressed on immune, infected and tumor cells (Nirschl and Drake, 2013), and, PD-1 expressed on T cells (Sakaguchi et al., 2008; Fife et al., 2009; Francisco et al., 2010; Nirschl and Drake, 2013; Schietinger and Greenberg, 2014; Bardhan et al., 2016; Sharpe and Pauken, 2017).

SI-1.2 A TCR activation primer

For a strong CD8+ T cell activation, three well-known signals have to be provided from professional antigen presenting cells (APCs) (Kindt et al., 2007):

- Signal 1: Antigen presentation to T cells.
- Signal 2: Co-stimulation.
- Signal 3: Cytokine priming.

Signal 1 is mediated by binding of a T cell receptor (TCR) on T cells with its cognate antigen presented on an MHC.

Signal 2 is mediated by a series of receptor:ligand bindings, such as CD80 binding to CD28 between the APC and the T cell, respectively. The combination of TCR engagement, CD28 binding, and IL-2 activates Zap-70, lck and PI3K, which in turn lead to T cell activation, expansion, and acquisition of effector activities (Liechtenstein et al., 2012; Rendall and Sontag, 2017). Furthermore, in reality, as Liechtenstein et al. (2012) states, a variety of ligand-receptor interactions take place in the immunological synapse, many of which are inhibitory. The final integration between activatory and inhibitory interactions determine the type and strength of the co-stimulatory signal given to the T cells, setting the "degree" of T cell activation.

Signal 3 is mediated by binding of cytokines to their respective receptors, such as IL-2 produced by T cells binding to IL-2 receptors also on the same T cells.

During antigen presentation to naïve T cells, PD-1:PD-L1 interaction acts as a brake in TCR signal transduction (Nirschl and Drake, 2013). PD-1 is transiently up-regulated during antigen presentation as a consequence of T cell activation (Freeman et al., 2000) and PD-1:PD-L1 binding results in ligand-induced TCR down-modulation (Escors et al., 2011; Karwacz et al., 2011, 2012).

To this end, Liechtenstein et al. (2012) suggest that TCR down-modulation is absolutely required for T cell activation in order to prevent T cell hyperactivation by terminating TCR signal transduction. In such cases, PD-1 associates to the TCR at the immunological synapse and controls its signal transduction as well

as its presence on the T cell surface (Karwacz et al., 2011). TCR down-modulation is largely reduced when PD-L1 is silenced in antigen-presenting DCs, or when PD-1:PD-L1 is blocked using antibodies during antigen presentation (Liechtenstein et al., 2012).

Finally, as further reviewed in (Liechtenstein et al., 2012), PD-1:PD-L1 interactions control the timing of TCR stimulation in at least two different ways: (*i*) by removing TCRs from the T cell surface, and (*ii*) by terminating the intracellular signal transduction pathways after recruiting phosphatases SHP1 and SHP2 (Zhang and Rundell, 2006; Bardhan et al., 2016). Note briefly that processes in (*ii*), put, for example, a brake on NF-κB signaling, the inhibitory process that shuts down IRF4, which in turn removes the Blimp-1 imposed brake from PD-1 transcription, where both IRF4 and Blimp-1 are key molecular species of our analysis, as discussed below in detail.

SI-1.3 Linking observations with immunological mechanisms

(O1) Anti-tumor CD8+ T cells are shunted to the lung during influenza infection.

Here, we discuss six mechanisms (O1-M1) - (O1-M6). In order to get insight into mechanism (O1-M1), we subdivide mechanism (O1-M1-B) into two complementary immunological mechanisms (O1-M1-A) and (O1-M1-B).

Mechanism (O1-M1-A) Cytotoxic CD8+ T cells ($\mathrm{T_{EFF}}$) are highly dynamic within dense tissue once activated, immediately followed by their migration arrest, induced by integrin upregulation that stops motion and promotes effective synapse formation (Feinerman et al., 2008) when contacting a high potency antigen available at adequate density on tissue resident antigen-presenting cells (APCs) (Marelli-Berg et al., 2010; Honda et al., 2014). Indeed, T cells in tissues migrate along chemotactic gradients until they encounter antigen on an APC, which leads to their Intercellular Adhesion Molecule (ICAM-1)-dependent arrest by TCR-mediated “stop signals” (Jennrich et al., 2012).

Specifically, activated T cells have been shown to rapidly traffic in tissue in response to chemokines and cytokines (Kindt et al., 2007; Chimen et al., 2017), including CXCL9, CXCL10, INFγ, *etc.* (Ogawa et al., 2002; Baaten et al., 2013; Oelkrug and Ramage, 2014; Kim and Chen, 2016; Spranger, 2016; Stein et al., 2016) with a primary function to find and kill target cells expressing cognate antigen (Ag) (Bhat et al., 2014).

Dynamic speed and travel patterns of $\mathrm{T_{EFF}}$ are predominantly influenced by the tissue environment rather than by mechanisms intrinsic to the $\mathrm{T_{EFF}}$. Specifically, activated T cells have been shown *in vivo* to traffic to inflamed skin, even in the absence of cognate Ag (Biotec and Gladbach, 2011), suggesting that Ag alone does not play an essential role in the recruitment of circulating CD8+ T cells (Van Braeckel-Budimir and Harty, 2017).

To this end, a natural question arises: “Why is it the anti-tumor $\mathrm{T_{EFF}}$ cells that are shunted to the infected lung, and not vice versa, that is, why is it not the anti-influenza $\mathrm{T_{EFF}}$ cells that are shunted to the tumor compartment instead?”

Mechanism (O1-M1-B) The above question can be addressed by reviewing *in vivo* studies describing T cell *Ag-induced arrest* in tissues in direct proportion to the amount of Ag present (Beattie et al., 2010; Deguine et al., 2010; Celli et al., 2011; Honda et al., 2014). Indeed, the time needed for the $\mathrm{T_{EFF}}$ killing of highly antigenic cells *in vivo* through the cell-to-cell attachment and TCR-pMHC (Ag) binding events can be partiality attributed to effective half-life or “confinement time” of a TCR-pMHC interaction (Aleksic et al., 2010).

The process can last for long periods of time depending on the context, (*i*) in the range of 40 minutes, (*ii*) over 3-6 hours and (*iii*) up to 48 hours, in order to form "stable immunologic synapses" (Grakoui et al., 1999; Liechtenstein et al., 2012; Xie et al., 2013; Tkach and Altan-Bonnet, 2013; Ortega-Carrion and Vicente-Manzanares, 2016; Stein et al., 2016), which are needed to complete a series of signaling events, including co-receptor recruitment and TCR phosphorylation (McKeithan, 1995; Garcia et al., 2007; Breart et al., 2008; Tkach and Altan-Bonnet, 2013; Liu et al., 2014; Das et al., 2015; Parello and Huseby, 2015).

Although T cells become rapidly arrested after their contact with Ag-presenting tissue-resident cells, their subsequent recovery period was found to be heterogeneous, with some cells regaining motility within 30 min and others remaining arrested for several hours (Honda et al., 2014). After the extended arrest, T cells can be oscillating between periods of brief arrest and motility, suggestive of additional TCR stimulation, before regaining a migration pattern similar to what was observed in the absence of antigen (Honda et al., 2014).

Such Ag-induced arrested T cells are functionally very distinct of highly motile T cells, because the arrested T cells were found to be characterized by a profoundly increased production of INFγ (Honda et al., 2014).

All this suggests that T cell Ag-induced arrest on target cells positively correlates with their effector function, and the balance between motility and Ag-induced arrest controls T cell activation (Stein et al., 2016).

Here, the definitions of "long periods of time" and "stable immunologic synapses" should be understood dynamically and not statically in terms of "fast association and dissociation rates" (Coombs et al., 2011; Tkach and Altan-Bonnet, 2013) that define the "confinement time" of a TCR-pMHC interaction (Aleksic et al., 2010). Multiple studies have indicated that T cells integrate these discontinuous antigen contacts over time and respond in proportion to the cumulative duration of TCR signaling as reviewed in (Tkach and Altan-Bonnet, 2013).

Many tumor-specific antigens provoke only weak immune responses, which are incapable of eliminating all tumor cells (Aleksic et al., 2010). This is in line with the McKeithan-Altan-Bonnet-Germain kinetic proofreading model (KPL-IFFL) (Hopfield, 1974; McKeithan, 1995; Altan-Bonnet and Germain, 2005; François et al., 2013; Courtney et al., 2017), which is based on the well-established fact that T cell activation is selected by evolution to discriminate a few foreign peptides rapidly from a vast excess of self-peptides (François et al., 2013). We use the abbreviation KPL-IFFL for the kinetic proofreading model coupled with limited signaling and incoherent feedforward loop (Lever et al., 2016).

Because many tumor antigens are self antigens (Liechtenstein et al., 2012), often called Tumour-Associated Antigens (TAAs) and Tumor-Specific Antigens (TSA) (Kindt et al., 2007), anti-tumor TCRs may be of lower affinity due to their selection and training against "self"-antigen reactivity (Hogquist and Jameson, 2014) compared with those TCRs evolved to recognize viral epitopes (Irving et al., 2012; Vonderheide and June, 2014). Indeed, the affinity of TCR clones for novel or not previously encountered antigens, like tumor antigens, is remarkably low, typically 1 - 10 μM (Courtney et al., 2017). This is in line with a commonly accepted fact that high-affinity tumour-specific TCR clonotypes are typically deleted from the available repertoire during thymic selection because the vast majority of targeted epitopes are derived from autologous proteins (Tan et al., 2015).

This phenomenon is known as "antigen discrimination" (Galvez et al., 2016; Rendall and Sontag, 2017), and is currently discussed in terms of the "antigen-receptor (catch bonds) lifetime dogma" (Feinerman et al., 2008; François and Altan-Bonnet, 2016).

In addition to the TCR antigen discrimination, tumors do not express large amounts of cognate Ag on MHCs to keep systemic tolerance and prevent the development of autoimmune diseases (Liechtenstein et al., 2012; Nirschl and Drake, 2013), compared with viral infection.

Antigen expression by tumor cells thus determines $\mathrm{T_{EFF}}$ motility within the tumor (Boissonnas et al., 2007). Such mobile $\mathrm{T_{EFF}}$ cells can follow collagen fibers or blood vessels, or migrate along blood vessels preferentially adopting an elongated morphology (Boissonnas et al., 2007), when nothing can prevent them from freely moving along high infection-induced chemokine gradients toward the infected and inflamed lung.

We finalize the description of this mechanism by pointing out a very important and relevant study (Poleszczuk et al., 2016) which documents intensive motility of anti-timor $\mathrm{T_{EFF}}$ cells when the anti-timor $\mathrm{T_{EFF}}$ cells enter and leave the TME back to the bloodstream and lymph multiple times before they get finally arrested and absorbed by the multiple-time revisited TME.

Mechanism (O1-M2) Tumors themselves may induce emigration of tumor-specific CD8+ T cells from the TME by employing chemokines like SDF-1/CXCL12 (Marelli-Berg et al., 2010; Joyce and Fearon, 2015; Kim and Chen, 2016). When the level of CXCL12 becomes greater than the levels of other chemoattractors, CXCL12 acts as chemorepeller (Marelli-Berg et al., 2010; Vianello et al., 2005), that is, at low levels, CXCL12 is a chemoattractor, while at high levels CXCL12 is a chemorepeller. Expression of CXCL12 and its receptor CXCR4 is induced by IFNγ (Ogawa et al., 2002; de Oliveira et al., 2013).

Sometimes, this effect is called *fugetaxis* (Vianello et al., 2005). Because IFNγ is produced in large quantities by different cell types in inflamed infected sites (Kindt et al., 2007), and then circulates to the tumor site within the bloodstream, it can be concluded that (distant) infection can significantly contribute to the egress of anti-tumor CD8+ T cells from the TME.

Mechanism (O1-M3) Due to non-specific cardiovascular edema effects, caused by infection-induced inflammation (Marchuk, 1997), the general circulation pattern of central memory ($\mathrm{T_{CM}}$) and naïve T cells (Donnadieu, 2016; Levin et al., 2016) throughout the body from blood, across high endothelial venules (HEVs) into lymph nodes, through T cell zones, out via efferent lymphatics, and eventually back into the blood through the thoracic duct is significantly perturbed and is redirected to the site of infection-induced inflammation (Marchuk, 1997; Levin et al., 2016).

Mechanism (O1-M4) As mentioned earlier, different cells in infected tissues induce cytokine production (Kindt et al., 2007). Cytokines play multiple roles such as chemoattraction of dendritic cells, macrophages, T cells, NK cells, and promotion of T cell adhesion to endothelial cells (Dufour et al., 2002). To this end, significant levels of both activated influenza-specific and non-specific T cells were found present in infected lung and measured (Toapanta and Ross, 2009).

The inflammatory chemokine receptor CXCR3 has been recently identified with effective T cell function. CXCR3 expression is increased during T cell activation and is important for homing to inflammatory sites. Its ligands CXCL9, CXCL10 and CXCL11 are rapidly induced during inflammation and guide T cells into specific microenvironments in lymphoid and non-lymphoid tissues as reviewed in (Stein et al., 2016).

Mechanism (O1-M5) High levels of IL-2 produced by activated anti-infection CD8+ T cells at the infection site counteract the repelling action of CXCL12 (Beider et al., 2003) in contrast to the opposite effect elicited by CXCL12 in the TME as discussed earlier.

Mechanism (O1-M6) The PD-1 mediated control of immune responses depends on interactions between PD-1 on CD8+ T cells and PD-L1 in tissues (Nirschl and Drake, 2013), inducing CD8+ T cell motility

paralysis via PD-1:PD-L1 stable bonds (Zinselmeyer et al., 2013; Schietinger and Greenberg, 2014; Stein et al., 2016). We introduce the "paralysis" mechanism **(O1-M6)** into the context of our studies by discussing systematically the following specific questions,

(Q.1) "What triggers expression of PD-1 receptors on CD8+ T cells, and why is the expression triggered in the first place?"

(Q.2) "Why are PD-1 receptors over-expressed in larger quantities on anti-tumor CD8+ T cells and not on anti-influenza CD8+ T cells co-localized within the same infected lung?"

(Q.3) "Why are anti-VACV CD8+ T cells not sequestered in the infected lung when the host is distantly co-infected with both infections, influenza A and VACV infections (in the absence of tumors)?"

To address (Q.1), we follow Simon and Labarriere (2017) who reviewed results highlighting the ambiguous role of PD-1 in defining efficient or inefficient adaptive immune response. Initially, PD-1 transient expression on native T cells is induced immediately upon TCR activation, that is, the number of PD-1 receptors can be regarded as a biomarker of activated and *not* exhausted CD8+ T cells.

The level of PD-1 receptors decreases in the absence of TCR signaling but is maintained upon chronic activation with a persistent epitope target such as in chronic viral infections and in cancer (Wherry et al., 2007; Brown et al., 2010; Pauken and Wherry, 2015b,a). Thus, the number of PD-1 receptors can *also* be regarded as a biomarker of exhausted T cells (Simon and Labarriere, 2017). Importantly, transient expressions of PD-1 and PD-L1 is viewed as a window of opportunity in the combined radiation (RT) and anti-PD-1:PD-L1 therapies (Kosinsky et al., 2018).

The discussed ambiguous role of PD-1, which can be viewed either as a biomarker of activated or exhausted CD8+ T cells depending on the inflammation context, can be explained as follows.

First, although the central immune tolerance mechanism results in the removal of most of the auto- or self-reactive T cells during thymic selection, a fraction of self-reactive lymphocytes escapes to the periphery and poses the threat of autoimmunity. Moreover, "it is now understood that the T cell repertoire is in fact broadly self-reactive, even self-centered" (Hogquist and Jameson, 2014; Grossman and Paul, 2015; Richards et al., 2016).The strength with which a T cell reacts to self ligands and the environmental context in which this reaction occurs influence almost every aspect of T cell biology, from development to differentiation to effector function (Hogquist and Jameson, 2014; Grossman and Paul, 2015).

The immune system has evolved various mechanisms to constrain autoreactive T cells and maintain peripheral tolerance (Grossman and Paul, 2001, 2015), including the constitutive expression of PD-L1 in large quantities in various tissues (*e.g.*, lungs, pancreatic islets, *etc.*), and T cell anergy, deletion, and suppression by regulatory T cells (Sakaguchi et al., 2008; Fife et al., 2009; Francisco et al., 2010; Schietinger and Greenberg, 2014; Bardhan et al., 2016).

Second, although T cells endow their host with a defense that favors pathogen clearance, this efficiency sometimes gives rise to intolerable immunopathology, especially when a pathogen transitions into a state of persistence. For this reason, the immune system is equipped with dampening mechanisms that induce T cell exhaustion via PD-1 and PD-L1 immune regulators (Zinselmeyer et al., 2013; Pauken and Wherry, 2015b; Bardhan et al., 2016). This means that the activated T cells must be attenuated irrespective of whether invaders are eliminated or persist. This is because, quite often, persisting microorganisms may cause less tissue damage than the associated immunopathology as a result of continued lymphocyte cytotoxicity (Speiser et al., 2016).

Overall, this means that the immune system prefers to put infection that cannot be eradicated rapidly into a chronic state which should produce less damage to the body than the extended exposition of the body to aggressive CD8+ T cell response (Grossman and Paul, 1992, 2015).

(O2) Disruption of anti-tumor responses is not due to tumor-induced immune suppression of viral clearance or the inability of the immune system to respond to concomitant challenges.

The observation (Kohlhapp et al., 2016) that cancer does not significantly suppress the natural anti-viral response can be explained by similar arguments used to introduce the mechanisms **(O1-M1)** - **(O1-M6)**. These suggest that much weaker inflammation in the tumor site compared with much stronger inflammation in the infected lung may not be enough to force the anti-influenza CD8+ T cells (arrested in the lung as discussed earlier) to egress the lung. The observation (Kohlhapp et al., 2016) that influenza infection did not alter the natural clearance of the VACV or the proportion of VACV-tetramer+ CD8+ T cells at the site of influenza infection can also be explained by the local anti-VACV CD8+ T cells Ag-induced arrest required to kill VACV-infected cells as discussed earlier.

(O3) Therapeutic blockade of PD-1 results in reversal of infection-mediated anti-tumor response disruption.

Recall that PD-L1 promotes motility paralysis (Zinselmeyer et al., 2013; Schietinger and Greenberg, 2014; Stein et al., 2016). In other words, the bond PD-1:PD-L1 mediates locking T cells into a state of prolonged motility paralysis by localizing to the environment with abundant PD-L1 expression on stromal cells as discussed earlier, termed “T cell motility paralysis” in (Zinselmeyer et al., 2013; Schietinger and Greenberg, 2014).

Because the bond PD-1:PD-L1 is formed dynamically due to interchanging binding and unbinding processes, blockade of PD-1 shifts the dynamic equilibrium towards dissociation of PD-1:PD-L1 bond, leading to the rapid recovering (about 30 min.) of T cell motility, signaling, and cytokine production (Zinselmeyer et al., 2013; Oelkrug and Ramage, 2014; Pauken et al., 2016). The corresponding details are summarized in Table SI-1.1 of Sect. SI-1.4.

Reactivated anti-tumor CD8+ T cells then detach from local PD-L1 anchors and start moving with lymph outside of the infected lung and may ultimately return back to the tumor site (Calzascia et al., 2005) with the blood flow, following similar trafficking routes and mechanisms as discussed in (Poleszczuk et al., 2016).

SI-1.4 Reactivation of exhausted effector cells

Because the PD-1 blockade reactivates exhausted anti-tumor CD8+ T cells, sequestered in the infected lung and return, possibly, them back to the TME (Kohlhapp et al., 2016), we briefly summarize relevant known results on the exhausted T cell reactivation (Table SI-1.1). Our summary is based on the recent reports (Zinselmeyer et al., 2013; Pauken et al., 2016; Wang et al., 2017).

Several gene signatures based on the analyses of populations of dysfunctional CD8+ T cells from cancer and chronic viral infections have been published and reviewed by Wang et al. (2017). These signatures confirm great similarity between virus- and cancer-associated CD8+ T cell dysfunction. Due to these published gene signature comparisons, we believe that Table SI-1.1 further supports mechanisms formulated in the main text.

Table SI-1.1. A brief summary of T_{EX} reactivation after PD-1 blockade.

Reactivation Effect	Mechanisms
**(E.1) reactivation of $\mathbf{T_{EFF}}$ functions in $\mathbf{T_{EX}}$:**	
(E.1.1) *improved cell cycle and proliferation*	• increased transcription of cell division genes<br>• increased levels of Ki-67
(E.1.2) *improved response to antigen*	• elevated co-production of INFγ and TNFα
(E.1.3) *improved motility and chemotaxis*	• upregulated expression of *cxcl9* and *cxcr3*
(E.1.4) *improved killing capability*	• increased levels of granulocytes
(E.1.5) *suppression of PD-1 expression*	• upregulated expression of *prdm1* encoding Blimp-1
(E.1.6) *protection against exhaustion*	• upregulated expression of *il7r*<br>• OCR state specific regulation of *ctla4*
**(E.2) reactivation of $\mathbf{T_{MEM}}$ functions in $\mathbf{T_{EX}}$:**	
(E.2.1) *negative regulation of apoptosis*	• increased levels of phospho-STAT5
(E.2.2) *improved adhesion*	• unknown
(E.2.3) *improved regulation of activation*	• elevated production of INFγ
**(E.3) transient reinvigoration of $\mathbf{T_{EX}}$** (peaked in 3-weeks)	
transient and Ag-dose dependent expression of prdm1 encoding Blimp-1	• small and large amounts of Ag repress *prdm1*<br>• medium amounts of Ag activate *prdm1*
**(E.4) signaling and immunometabolic effects:**	
(E.4.1) *signaling*	• upregulation of genes encoding NF-κB and IRFs
(E.4.2) *lipid metabolism*	• upregulation of genes encoding PPARγ and RXRα<br>• downregulation of *srebp1*
(E.4.3) *de-novo cholesterol pathway, and glycolysis*	• unknown

SI-2 A CORE MATHEMATICAL MODEL OF PD-1 EXPRESSION

SI-2.1 The model equations

Our core mathematical model of PD-1 expression on the surface of a CD8+ T cell describes normal and aberrant dynamics of interactions between four immunobiochemical entities, Bcl-6 (C), PD-1 (P), IRF4 (I), and Blimp-1 (B),

$$\underbrace{\frac{dC}{dt}}_{C=[\text{Bcl6}]} = \underbrace{\left(\frac{a_c U^{n_c}}{A_c^{n_c} + U^{n_c}}\right)}_{\text{TCR dep. act.}} \underbrace{\left(\frac{M_c^{r_c}}{M_c^{r_c} + B^{r_c} + I^{r_c} + C^{r_c}}\right)}_{\text{Blimp1/IRF4/Bcl6 dep. repr.}} - \underbrace{\mu_c C,}_{\text{Bcl6 deg.}} \tag{SI-2.1a}$$

$$\underbrace{\frac{dP}{dt}}_{P=[\text{PD1}]} = \left(\sigma_p + \underbrace{\frac{a_p U^{n_p}}{A_p^{n_p} + U^{n_p}}}_{\text{TCR dep. act.}}\right) \underbrace{\left(\frac{M_p^{r_p}}{M_p^{r_p} + B^{r_p}}\right)}_{\text{Blimp1 dep. repr.}} - \underbrace{\mu_p P,}_{\text{PD1 deg.}} \tag{SI-2.1b}$$

$$\underbrace{\frac{dI}{dt}}_{I=[\text{IRF4}]} = \left(\sigma_i + \underbrace{\frac{a_i U^{n_i}}{A_i^{n_i} + U^{n_i}}}_{\text{TCR dep. act.}} + \underbrace{\frac{k_i B^{m_i}}{K_i^{m_i} + B^{m_i}}}_{\text{Blimp1 dep. act.}} + \underbrace{\frac{q_i I^{s_i}}{Q_i^{s_i} + I^{s_i}}}_{\text{IRF4 dep. act.}}\right) \Phi_{\text{L}} - \underbrace{\mu_i I,}_{\text{IRF4 deg.}} \tag{SI-2.1c}$$

$$\underbrace{\frac{dB}{dt}}_{B=[\text{Blimp1}]} = \left(\underbrace{\frac{a_b U^{n_b}}{A_b^{n_b} + U^{n_b}}}_{\text{TCR dep. act.}} + \underbrace{\frac{k_b I^{m_b}}{K_b^{m_b} + I^{m_b}}}_{\text{IRF4 dep. act.}}\right) \underbrace{\left(\frac{M_b^{r_b}}{M_b^{r_b} + C^{r_b}}\right)}_{\text{Bcl6 dep. repr.}} - \underbrace{\mu_b B.}_{\text{Blimp1 deg.}} \tag{SI-2.1d}$$

Here, for the sake of compactness in the equation term explanation, we use the following abbreviations, “TCR dep. act.” for TCR-dependent activation, “Blimp-1/IRF4/Bcl-6” for Blimp-1/IRF4/Bcl-6-dependent repression, and so on.

The model structure corresponds to the circuit topology depicted in Fig. 2 of the main text with a few simplifications resulting from lumping some species, (*i*) NFATc1 and PD-1 becoming the species P, and (*ii*) NF-$\varkappa$B and IRF4 becoming the species I. We also omit Erk-dependent degradation of Bcl-6 because it is in turn attenuated by Bcl-6 itself.

The input $U := U(\alpha, \kappa, P)$ to the model (SI-2.1) is described by the scalar function $u(\alpha, \kappa)$ defined in (SI-3.11),

$$U(\alpha, \kappa, P) = u(\alpha, \kappa)\, \phi_L(P), \tag{SI-2.2a}$$

$$\phi_L(P) = \frac{H_p}{H_p + L\,P}. \tag{SI-2.2b}$$

Here, the inhibitory regulatory factor $\phi_L(P)$ corresponds to the co-localization of PD-1:PD-L1 complexes around the immunologic Ag-TCR synapses that hinder the TCR activity as discussed in Sec. SI-1.2. An external environment parameter L models a fraction of PD-1 receptors bound with PD-L1. Parameters α and κ are scaled Ag level and scaled k_{off}, the dissociation constant for the Ag-TLR bond, respectively.

We use a Michaelis-Menton saturation functional dependence in the expression (SI-2.2b) to describe a 2D-sliding diffusion of PD-1 receptors on the surface of a T cell (without any switch-like reaction sharp transitions) as a major process contributing to the TCR down regulation effect (Sect. SI-1.2).

Next, the factor $\Phi_{\mathrm{L}} := \Phi_{\mathrm{L}}(P)$ in the equation (SI-2.1c) describes a net negative feedback effect caused by the PD-1:PD-L1 interaction (Sec. SI-1.2),

$$\Phi_{\mathrm{L}}(P) = \frac{H_L^{h_L}}{H_L^{h_L} + (L\,P)^{h_L}}. \tag{SI-2.3}$$

Recall that the active complex formed between PD-1 and PD-L1 suppresses the NF-κB pathway, while the NF-κB pathway activates IRF4 (Fig. 2).

We also use generic Hill functions in (SI-2.2b) and (SI-2.3) following the Hill-function approximation suggested for T cell exhaustion in (Johnson et al., 2011).

In order to capture effects caused by self (tumor) and non-self (infection) interactions, including significant differences in the magnitude of infection and amount of tumor antigens, we implement the following relationships to mathematically implement the self- / non-self specificity,

$$\alpha_{\mathrm{T}} < \alpha_{\mathrm{I}}, \tag{SI-2.4a}$$

$$\kappa_{\mathrm{T}} > \kappa_{\mathrm{I}}. \tag{SI-2.4b}$$

Here, subscript labels “T” and “I” correspond to tumor and infection, respectively.

Based on our immunobiochemical reconstruction and following (Warmflash and Dinner, 2009), we make explicitly additional choices to rank TCR-mediated activation parameters as follows,

$$A_c \leq A_p \leq A_i \leq A_b, \tag{SI-2.5a}$$

$$a_c \leq a_p \leq a_i \leq a_b. \tag{SI-2.5b}$$

Based on the developed immunobiochemical reconstruction, the inequality choices (SI-2.5a) ensure that the genes encoding Bcl-6 and PD-1 are activated at lower antigen levels than the genes encoding IRF4 and Blimp-1, whereas the latter ensures that the switch towards the suppression of PD-1 transcription is biased towards the CD8+ T cell, when both IRF4 and Blimp-1 are expressed at high antigen levels.

To account for the abundance of the lumped TNFα/IFNγ species, we have replaced the rate constant σ_p in the equation (SI-2.1b) by the reaction rate expression,

$$\tilde{\sigma}_p = \sigma_p + \frac{k_{\mathrm{T}} T^{n_T}}{K_T^{n_T} + T^{n_T}}. \tag{SI-2.6}$$

Here, T corresponds to TNFα, $k_T = 0.5$, $k_T = 1$, and $n_T = 2$. The values of the new parameters are selected in the range of the corresponding parameter values from Table SI-2.1.

SI-2.2 The model parameters

Reference parameter values used in our modeling studies are listed in Table SI-2.1. We have to mention explicitly that the parameter values have not been fitted to any data from (Kohlhapp et al., 2016), and have been selected as follows.

Table SI-2.1. PD-1 expression model parameter values.

Parameters	Values	Comments
σ_i	0.30	IRF4 constituent synthesis rate
σ_p	0.10	PD-1 immune central tolerance const. synth. rate
$a_c = a_p < a_i < a_b$	$0.75 = 0.75 < 75.0 < 100.00$	genetic switch thresholds
$A_c < A_p < A_i < A_b$	$0.01 < 0.10 < 1.00 < 10.00$	genetic switch thresholds
$n_c = n_p$	3	species: Bcl-6 and PD-1
$n_b = n_i$	2	species: Blimp-1 and IRF4
k_b	$0 - 25$	species: IRF4
$k_i = q_i$	7.50	species: Blimp-1 and IRF4
$K_b = K_i = Q_i$	1.00	species: Blimp-1 and IRF4
$m_b = m_i = s_i$	2	species: Blimp-1 and IRF4
$M_b = M_c = M_p$	10.00	species: Blimp-1, Bcl-6 and PD-1
$H_p = H_L$	0.1	species: PD-1 and PD-L1
$r_b = r_c$	2	species: Blimp-1 and Bcl-6
$r_p = h_L$	4	species: PD-1 (p) and PD-L1 (L)
$\mu_c = \mu_p$	0.10	species: Bcl-6 and PD-1
$\mu_b = \mu_i$	1.00	species: Blimp-1 and IRF4
L	0 - 1	species: fraction of PD-1 bound to PD-L1

First, we used dimensionless (scaled) parameter values of the same order of magnitude for the corresponding subsets of parameters as those which were used in (Sciammas et al., 2011; Martinez et al., 2012; Lever et al., 2016).

In our selection of the reference parameter values (Table SI-2.1), we also analyzed and followed a number of insightful discussions of a very challenging and complex problem of selecting relevant parameter values for biological and especially immunological models, presented in a number of published works (Heinrich and Rapoport, 2005; Warmflash and Dinner, 2009; Martinez et al., 2012; Lever et al., 2014; Galvez et al., 2016), including conceptual views (Gunawardena, 2014; Eftimie et al., 2016) as well as discussed general issues with experimental measurements (De Boer and Perelson, 2013; Eftimie et al., 2016).

Second, the parameter values used from (Sciammas et al., 2011; Lever et al., 2016) can be justified for our modeling studies by employing the following IFFL function argument. Indeed, the incoherent feedforward loops cannot exert their biphasic function with any arbitrary parameter values (Kim et al., 2008). The parameter values taken from (Sciammas et al., 2011; Lever et al., 2016) and used in the model (SI-2.1) correspond to the dose-dependent biphasic behaviors as defined and studied in (Kim et al., 2008), and also observed experimentally in the cited literature. In other words, the used parameter values are sufficient to instill the IFFL function.

Finally, the type of modeling carried out in our work can be characterized as phenotypic modeling (Warmflash and Dinner, 2009; Lever et al., 2014; Gunawardena, 2014). Recall that the objective of the phenotypic modeling is to capture the function of a biological system, based on the available and well-established features of the regulatory network under study as also explicitly stated in (Sciammas et al., 2011) which justified the selection of generic Hill functions in their model tailored to the GRN topology. In this work, we implemented a similar approach.

SI-3 THE KPL-IFFL MODEL

For the sake of consistency in the integration of the model (Lever et al., 2016) with our model describing the core circuit, we briefly derive functional relationships needed for the models' integration, adapting the discussion in (Lever et al., 2016).

Specifically, our objective here will be to derive the function $u(\alpha, \kappa)$, which we define as a non-dimensionalized input P in (SI-3.10b), and for which the final expression is given in (SI-3.11). The scaled function $u(\alpha, \kappa)$ depends on two state variables α and κ, the scaled level of Ag and the scaled value of the off-rate constant k_{off}, respectively.

A mathematical model (Lever et al., 2016) is

$$\frac{dL}{dt} = -k_{\text{on}}LR + k_{\text{off}}C_{\text{T}}, \tag{SI-3.1a}$$

$$\frac{dR}{dt} = -k_{\text{on}}LR + k_{\text{off}}C_{\text{T}}, \tag{SI-3.1b}$$

$$\frac{dC_0}{dt} = k_{\text{on}}LR - (k_{\text{off}} + k_{\text{p}})\, C_0, \tag{SI-3.1c}$$

$$\frac{dC_1}{dt} = k_{\text{p}}C_0 - (k_{\text{off}} + k_{\text{i}})\, C_1, \tag{SI-3.1d}$$

$$\frac{dC_2}{dt} = k_{\text{i}}C_1 - k_{\text{off}}C_2, \tag{SI-3.1e}$$

$$\frac{dY}{dt} = \gamma_+^y\,(Y_{\text{T}} - Y) - \gamma_-^y Y + \lambda C_1\,(Y_{\text{T}} - Y)\,, \tag{SI-3.1f}$$

$$\frac{dP}{dt} = \gamma_+^p\,(P_{\text{T}} - P) - \gamma_-^p P + \delta Y\,(P_{\text{T}} - P) - \mu C_1 P. \tag{SI-3.1g}$$

The state variables and parameters of the model (SI-3.1) are defined in (Lever et al., 2016). Parameters important for our derivation are: k_{on} and k_{off} are on- and off-rate constants, k_{p} is the kinetic proofreading rate constant, k_{i} is the kinetic rate constant for transforming of the active complex C_1 into the inactive complex C_2. We will also need C_{T}, the total number of all ligand-receptor complexes,

$$C_{\text{T}} = C_0 + C_1 + C_2. \tag{SI-3.2}$$

Here, C_{T} does not correspond to any conserved moiety and, instead, changes in time.

The model (SI-3.1) has the following first integrals, also termed moiety conservation relationships,

$$L_{\text{T}} = L + C_{\text{T}}. \tag{SI-3.3a}$$

$$R_{\text{T}} = R + C_{\text{T}}, \tag{SI-3.3b}$$

Due to the relationships (SI-3.3b) and (SI-3.3a), the corresponding first two equations (SI-3.1a) and (SI-3.1b) in the model (SI-3.1) become redundant and are omitted from further analysis.

Setting the model linearly independent equations (SI-3.1c) - (SI-3.1g) at steady state, we can obtain the following algebraic relationships,

$$C_0 = \left(\frac{k_{\mathrm{on}}}{k_{\mathrm{off}} + k_{\mathrm{p}}}\right) \times LR, \tag{SI-3.4a}$$

$$C_1 = \left(\frac{k_{\mathrm{p}}}{k_{\mathrm{off}} + k_{\mathrm{i}}}\right) \times C_0, \tag{SI-3.4b}$$

$$C_2 = \left(\frac{k_{\mathrm{i}}}{k_{\mathrm{off}}}\right) \times C_1, \tag{SI-3.4c}$$

$$Y = \left(\frac{1 + \left(\lambda/\gamma^y_+\right) C_1}{1 + \left(\gamma^y_-/\gamma^y_+\right) + \left(\lambda/\gamma^y_+\right) C_1}\right) \times Y_{\mathrm{T}}, \tag{SI-3.4d}$$

$$P = \left(\frac{1 + \left(\delta/\gamma^p_+\right) Y}{1 + \left(\gamma^p_-/\gamma^p_+\right) + \left(\mu/\gamma^p_+\right) C_1 + \left(\delta/\gamma^p_+\right) Y}\right) \times P_{\mathrm{T}}. \tag{SI-3.4e}$$

Next, we eliminate the product LR from (SI-3.4a) by using (SI-3.4a) - (SI-3.4c) in (SI-3.2),

$$C_{\mathrm{T}} = \left(\left(\frac{k_{\mathrm{on}}}{k_{\mathrm{off}} + k_{\mathrm{p}}}\right) + \left(\frac{k_{\mathrm{p}}}{k_{\mathrm{off}} + k_{\mathrm{i}}}\right)\left(\frac{k_{\mathrm{on}}}{k_{\mathrm{off}} + k_{\mathrm{p}}}\right) + \left(\frac{k_{\mathrm{i}}}{k_{\mathrm{off}}}\right)\left(\frac{k_{\mathrm{p}}}{k_{\mathrm{off}} + k_{\mathrm{i}}}\right)\left(\frac{k_{\mathrm{on}}}{k_{\mathrm{off}} + k_{\mathrm{p}}}\right)\right) LR. \tag{SI-3.5}$$

After simple algebraic manipulations, we obtain from (SI-3.5) that

$$LR = K_d C_{\mathrm{T}}, \quad K_d = \frac{k_{\mathrm{off}}}{k_{\mathrm{on}}}. \tag{SI-3.6}$$

Using (SI-3.6) in (SI-3.4a), and then (SI-3.4a) in (SI-3.4b), followed by using (SI-3.4b) in (SI-3.4c), we obtain

$$C_0 = \left(\frac{k_{\mathrm{off}}}{k_{\mathrm{off}} + k_{\mathrm{p}}}\right) C_{\mathrm{T}}, \tag{SI-3.7a}$$

$$C_1 = \left(\frac{k_{\mathrm{p}}}{k_{\mathrm{off}} + k_{\mathrm{i}}}\right)\left(\frac{k_{\mathrm{off}}}{k_{\mathrm{off}} + k_{\mathrm{p}}}\right) C_{\mathrm{T}}, \tag{SI-3.7b}$$

$$C_2 = \left(\frac{k_{\mathrm{i}}}{k_{\mathrm{off}}}\right)\left(\frac{k_{\mathrm{p}}}{k_{\mathrm{off}} + k_{\mathrm{i}}}\right)\left(\frac{k_{\mathrm{off}}}{k_{\mathrm{off}} + k_{\mathrm{p}}}\right) C_{\mathrm{T}}. \tag{SI-3.7c}$$

Note that C_{T} is still unknown in (SI-3.7). To compute C_{T}, we use an alternative expression for the product LR.

Indeed, we can obtain from (SI-3.3a) and (SI-3.3b) that $L = L_{\mathrm{T}} - C_{\mathrm{T}}$ and $R = R_{\mathrm{T}} - C_{\mathrm{T}}$, respectively. Now, using $LR = (L_{\mathrm{T}} - C_{\mathrm{T}})(R_{\mathrm{T}} - C_{\mathrm{T}})$ in (SI-3.6), we come to a closed quadratic equation with respect to C_{T},

$$(L_{\mathrm{T}} - C_{\mathrm{T}})(R_{\mathrm{T}} - C_{\mathrm{T}}) = K_d C_{\mathrm{T}}. \tag{SI-3.8}$$

Solving the quadratic equation (SI-3.8) with respect to C_T, we obtain two solutions, only one of which corresponds to the biologically meaningful condition, $C_\mathrm{T} = 0$ at $L_\mathrm{T} = 0$,

$$C_\mathrm{T} = \frac{1}{2}\left(R_\mathrm{T} + L_\mathrm{T} + K_d - \sqrt{(R_\mathrm{T} + L_\mathrm{T} + K_d)^2 - 4R_\mathrm{T}L_\mathrm{T}}\right). \tag{SI-3.9}$$

The solution (SI-3.9) also corresponds to the stable equilibrium in the system of linearly independent equations (SI-3.1c) - (SI-3.1g).

It is convenient to nondimensionalize the equilibrium solution of (SI-3.1) given by the expressions (SI-3.4a) - (SI-3.4e), and (SI-3.9) by scaling all state variables and parameters as follows,

$$c_\mathrm{T} = \frac{C_\mathrm{T}}{R_\mathrm{T}}, \quad c_k = \frac{C_k}{R_\mathrm{T}}, \quad k = 0, 1, 2, \tag{SI-3.10a}$$

$$y = \frac{Y}{Y_\mathrm{T}}, \quad u = \frac{P}{P_\mathrm{T}}, \tag{SI-3.10b}$$

$$K_\mathrm{p} = \frac{1}{R_\mathrm{T}}\left(\frac{k_\mathrm{p}}{k_\mathrm{on}}\right), \quad K_\mathrm{i} = \frac{1}{R_\mathrm{T}}\left(\frac{k_\mathrm{i}}{k_\mathrm{on}}\right), \tag{SI-3.10c}$$

$$\Gamma^y = \frac{\gamma_-^y}{\gamma_+^y}, \quad \Gamma^p = \frac{\gamma_-^p}{\gamma_+^p}, \tag{SI-3.10d}$$

$$\Lambda = \lambda\frac{R_\mathrm{T}}{\gamma_+^y}, \quad \Delta = \delta\frac{Y_\mathrm{T}}{\gamma_+^p}, \quad \Theta = \mu\frac{R_\mathrm{T}}{\gamma_+^p}, \tag{SI-3.10e}$$

$$\alpha = \frac{L_\mathrm{T}}{R_\mathrm{T}}, \quad \kappa = \frac{K_d}{R_\mathrm{T}}. \tag{SI-3.10f}$$

We obtain from the rescaled (SI-3.4e) that

$$u(\alpha, \kappa) = \frac{1 + \Delta\, y(\alpha,\kappa)}{1 + \Gamma^p + \Theta v(\kappa)\, c_\mathrm{T}(\alpha,\kappa) + \Delta\, y(\alpha,\kappa)}. \tag{SI-3.11}$$

In (SI-3.11), the functions $c_1(\alpha)$ and $y(\alpha)$ are obtained from the corresponding expressions (SI-3.4b) and (SI-3.4d) rescaled as discussed earlier,

$$y(\alpha,\kappa) = \frac{1 + \Lambda v(\kappa)\, c_\mathrm{T}(\alpha,\kappa)}{1 + \Gamma^y + \Lambda v(\kappa)\, c_\mathrm{T}(\alpha,\kappa)}, \tag{SI-3.12a}$$

$$c_\mathrm{T}(\alpha,\kappa) = \frac{1}{2}\left(1 + \alpha + \kappa - \sqrt{(1 + \alpha + \kappa)^2 - 4\alpha}\right), \tag{SI-3.12b}$$

$$v(\kappa) = \left(\frac{K_\mathrm{p}}{\kappa + K_\mathrm{i}}\right)\left(\frac{\kappa}{\kappa + K_\mathrm{p}}\right). \tag{SI-3.12c}$$

Reference values of parameters used in the expressions (SI-3.11) - (SI-3.12) are listed in Table SI-3.1. These values correspond to the values used to compute Fig. 3 in (Lever et al., 2016).

Table SI-3.1. KPL-IFFL model parameter values.

№	Parameter	Value
1.	K_i	10^{-3}
2.	K_p	10^{-2}
3.	Γ^y	5×10^2
4.	Γ^p	5×10^2
5.	Δ	5×10^3
6.	Θ	5×10^4
7.	Λ	10^4
8.	α	$10^{-4} - 10^4$
9.	κ	$10^{-4} - 10^2$

SI-4 ANALYSIS OF SPARSE VERSUS DENSE EXPERIMENTAL DATA

The main limitation of experimental data (Kohlhapp et al., 2016) is that the data is sparse. Yet, in spite of this limitation, by focusing on the phenotypes (A) and (B) schematically depicted in Fig. 8, our model semi-quantitatively fits a body of experimental data both discussed in the current literature and in (Kohlhapp et al., 2016) with very a small number of variables and parameters.

The topic of limitations imposed by the sparsity of experimental data has been widely discussed in the biological and especially immunological literature in the context of the applicability of such data in mathematical modeling (De Boer and Perelson, 2013; François et al., 2013; Gunawardena, 2014; Eftimie et al., 2016) to mention just a few references.

Small-scale models are highly interpretative (James et al., 2013; Ledzewicz and Schattler, 2017) and, here, we agree with the following citation: "Simplified models are sometimes more predictive than elaborate ones when data are sparse and have the added benefit of transparency" (François et al., 2013). An added benefit of smaller and more phenomenological models is that they have a small number of parameters, for which one may be able to find rough estimates from the literature. In contrast, large scale models have many parameters, most of which may not be available from the literature and, instead, should be fitted to data. Such models may be more powerful in accurate predictions at the (possible) expense of loosing interpretability (James et al., 2013).

SI-5 MATHEMATICAL AND NUMERICAL METHODS

The steady-state solutions of the models developed in this work, the solution stability (Sontag, 2013), as well as the parameter continuation of the steady-state solutions (Kuznetsov, 2013) have been studied numerically (Khibnik et al., 1993), using the command-line functionality of `matcont6p10`, a Matlab®-based Continuation Toolbox (Dhooge et al., 2008). MATLAB® Parallel Computing Toolbox was employed whenever possible. Finally, the color maps were generated using `varycolor.m`, a Matlab®-based function.

REFERENCES

Aleksic, M., Dushek, O., Zhang, H., Shenderov, E., Chen, J. L., Cerundolo, V., et al. (2010). Dependence of T cell antigen recognition on T cell receptor-peptide MHC confinement time. *Immunity* 32, 163–174

Altan-Bonnet, G. and Germain, R. N. (2005). Modeling T cell antigen discrimination based on feedback control of digital ERK responses. *PLoS Biol.* 3, e356

Baaten, B. J., Cooper, A. M., Swain, S. L., and Bradley, L. M. (2013). Location, location, location: the impact of migratory heterogeneity on T cell function. *Frontiers in immunology* 4

Bardhan, K., Anagnostou, T., and Boussiotis, V. A. (2016). The PD1:PD-L1/2 pathway from discovery to clinical implementation. *Front Immunol* 7, 550

Beattie, L., Peltan, A., Maroof, A., Kirby, A., Brown, N., Coles, M., et al. (2010). Dynamic imaging of experimental Leishmania donovani-induced hepatic granulomas detects Kupffer cell-restricted antigen presentation to antigen-specific CD8+ T cells. *PLoS pathogens* 6, e1000805

Beider, K., Nagler, A., Wald, O., Franitza, S., Dagan-Berger, M., Wald, H., et al. (2003). Involvement of CXCR4 and IL-2 in the homing and retention of human NK and NK T cells to the bone marrow and spleen of NOD/SCID mice. *Blood* 102, 1951–1958

Bhat, P., Leggatt, G., Matthaei, K. I., and Frazer, I. H. (2014). The kinematics of cytotoxic lymphocytes influence their ability to kill target cells. *PLoS ONE* 9, e95248

Biotec, M. and Gladbach, B. (2011). In vivo imaging of T-cell motility in the elicitation phase of contact hypersensitivity using two-photon microscopy. *J Investig Dermatol* 131, 977–979

Boissonnas, A., Fetler, L., Zeelenberg, I. S., Hugues, S., and Amigorena, S. (2007). In vivo imaging of cytotoxic T cell infiltration and elimination of a solid tumor. *Journal of Experimental Medicine* 204, 345–356

Breart, B., Lemaître, F., Celli, S., and Bousso, P. (2008). Two-photon imaging of intratumoral CD8+ T cell cytotoxic activity during adoptive T cell therapy in mice. *The Journal of clinical investigation* 118, 1390

Brown, K. E., Freeman, G. J., Wherry, E. J., and Sharpe, A. H. (2010). Role of PD-1 in regulating acute infections. *Curr. Opin. Immunol.* 22, 397–401

Calzascia, T., Masson, F., Di Berardino-Besson, W., Contassot, E., Wilmotte, R., Aurrand-Lions, M., et al. (2005). Homing phenotypes of tumor-specific CD8 T cells are predetermined at the tumor site by crosspresenting APCs. *Immunity* 22, 175–184

Celli, S., Albert, M. L., and Bousso, P. (2011). Visualizing the innate and adaptive immune responses underlying allograft rejection by two-photon microscopy. *Nature medicine* 17, 744–749

Chimen, M., Apta, B. H., and Mcgettrick, H. M. (2017). Introduction: T cell trafficking in inflammation and immunity. In *T-Cell Trafficking* (Springer). 73–84

Coombs, D., Dushek, O., and van der Merwe, P. A. (2011). A review of mathematical models for T cell receptor triggering and antigen discrimination. In *Mathematical Models and Immune Cell Biology* (Springer). 25–45

Courtney, A. H., Lo, W. L., and Weiss, A. (2017). TCR signaling: mechanisms of initiation and propagation. *Trends Biochem. Sci.*

Das, D. K., Feng, Y., Mallis, R. J., Li, X., Keskin, D. B., Hussey, R. E., et al. (2015). Force-dependent transition in the T-cell receptor β-subunit allosterically regulates peptide discrimination and pMHC bond lifetime. *Proc. Natl. Acad. Sci. U.S.A.* 112, 1517–1522

De Boer, R. J. and Perelson, A. S. (2013). Quantifying T lymphocyte turnover. *J. Theor. Biol.* 327, 45–87

de Oliveira, K. B., Guembarovski, R. L., Guembarovski, A. M. F. L., do Amaral, A. C. d. S., Sobrinho, W. J., Ariza, C. B., et al. (2013). CXCL12, CXCR4 and IFNγ genes expression: implications for proinflammatory microenvironment of breast cancer. *Clinical and Experimental Medicine* 13, 211–219

Deguine, J., Breart, B., Lemaître, F., Di Santo, J. P., and Bousso, P. (2010). Intravital imaging reveals distinct dynamics for natural killer and CD8+ T cells during tumor regression. *Immunity* 33, 632–644

Dhooge, A., Govaerts, W., Kuznetsov, Y. A., Meijer, H. G. E., and Sautois, B. (2008). New features of the software matcont for bifurcation analysis of dynamical systems. *Mathematical and Computer Modelling of Dynamical Systems* 14, 147–175

Donnadieu, E. (2016). *Defects in T Cell Trafficking and Resistance to Cancer Immunotherapy* (Springer)

Dufour, J. H., Dziejman, M., Liu, M. T., Leung, J. H., Lane, T. E., and Luster, A. D. (2002). IFN-gamma-inducible protein 10 (IP-10; CXCL10)-deficient mice reveal a role for IP-10 in effector T cell generation and trafficking. *J. Immunol.* 168, 3195–3204

Eftimie, R., Gillard, J. J., and Cantrell, D. A. (2016). Mathematical models for immunology: current state of the art and future research directions. *Bull. Math. Biol.* 78, 2091–2134

Escors, D., Bricogne, C., Arce, F., Kochan, G., and Karwacz, K. (2011). On the Mechanism of T cell receptor down-modulation and its physiological significance. *J Biosci Med* 1

Feinerman, O., Germain, R. N., and Altan-Bonnet, G. (2008). Quantitative challenges in understanding ligand discrimination by $\alpha\beta$ T cells. *Mol. Immunol.* 45, 619–631

Fife, B. T., Pauken, K. E., Eagar, T. N., Obu, T., Wu, J., Tang, Q., et al. (2009). Interactions between PD-1 and PD-L1 promote tolerance by blocking the TCR-induced stop signal. *Nat. Immunol.* 10, 1185–1192

Francisco, L. M., Sage, P. T., and Sharpe, A. H. (2010). The PD-1 pathway in tolerance and autoimmunity. *Immunol. Rev.* 236, 219–242

François, P. and Altan-Bonnet, G. (2016). The case for absolute ligand discrimination: modeling information processing and decision by immune T cells. *Journal of Statistical Physics* 162, 1130–1152

François, P., Voisinne, G., Siggia, E. D., Altan-Bonnet, G., and Vergassola, M. (2013). Phenotypic model for early T-cell activation displaying sensitivity, specificity, and antagonism. *Proc. Natl. Acad. Sci. U.S.A.* 110, E888–897

Freeman, G. J., Long, A. J., Iwai, Y., Bourque, K., Chernova, T., Nishimura, H., et al. (2000). Engagement of the PD-1 immunoinhibitory receptor by a novel B7 family member leads to negative regulation of lymphocyte activation. *J. Exp. Med.* 192, 1027–1034

Galvez, J., Galvez, J. J., and Garcia-Penarrubia, P. (2016). TCR/pMHC interaction: phenotypic model for an unsolved enigma. *Front Immunol* 7, 467

Garcia, Z., Pradelli, E., Celli, S., Beuneu, H., Simon, A., and Bousso, P. (2007). Competition for antigen determines the stability of T cell-dendritic cell interactions during clonal expansion. *Proc. Natl. Acad. Sci. U.S.A.* 104, 4553–4558

Grakoui, A., Bromley, S. K., Sumen, C., Davis, M. M., Shaw, A. S., Allen, P. M., et al. (1999). The immunological synapse: a molecular machine controlling T cell activation. *Science* 285, 221–227

Grossman, Z. and Paul, W. E. (1992). Adaptive cellular interactions in the immune system: the tunable activation threshold and the significance of subthreshold responses. *Proc. Natl. Acad. Sci. U.S.A.* 89, 10365–10369

Grossman, Z. and Paul, W. E. (2001). Autoreactivity, dynamic tuning and selectivity. *Curr. Opin. Immunol.* 13, 687–698

Grossman, Z. and Paul, W. E. (2015). Dynamic tuning of lymphocytes: physiological basis, mechanisms, and function. *Annu. Rev. Immunol.* 33, 677–713

Gunawardena, J. (2014). Models in biology: 'accurate descriptions of our pathetic thinking'. *BMC biology* 12, 29

Heinrich, R. and Rapoport, T. A. (2005). Generation of nonidentical compartments in vesicular transport systems. *J. Cell Biol.* 168, 271–280

Hogquist, K. A. and Jameson, S. C. (2014). The self-obsession of T cells: how TCR signaling thresholds affect fate 'decisions' and effector function. *Nat. Immunol.* 15, 815–823

Honda, T., Egen, J. G., Lammermann, T., Kastenmuller, W., Torabi-Parizi, P., and Germain, R. N. (2014). Tuning of antigen sensitivity by T cell receptor-dependent negative feedback controls T cell effector function in inflamed tissues. *Immunity* 40, 235–247

Hopfield, J. J. (1974). Kinetic proofreading: a new mechanism for reducing errors in biosynthetic processes requiring high specificity. *Proc. Natl. Acad. Sci. U.S.A.* 71, 4135–4139

Irving, M., Zoete, V., Hebeisen, M., Schmid, D., Baumgartner, P., Guillaume, P., et al. (2012). Interplay between T cell receptor binding kinetics and the level of cognate peptide presented by major histocompatibility complexes governs CD8+ T cell responsiveness. *J. Biol. Chem.* 287, 23068–23078

James, G., Witten, D., Hastie, T., and Tibshirani, R. (2013). *An Introduction to Statistical Learning*, vol. 112 (Springer)

Jennrich, S., Lee, M. H., Lynn, R. C., Dewberry, K., and Debes, G. F. (2012). Tissue exit: a novel control point in the accumulation of antigen-specific CD8 T cells in the influenza a virus-infected lung. *J. Virol.* 86, 3436–3445

Johnson, P. L., Kochin, B. F., McAfee, M. S., Stromnes, I. M., Regoes, R. R., Ahmed, R., et al. (2011). Vaccination alters the balance between protective immunity, exhaustion, escape, and death in chronic infections. *J. Virol.* 85, 5565–5570

Joyce, J. A. and Fearon, D. T. (2015). T cell exclusion, immune privilege, and the tumor microenvironment. *Science* 348, 74–80

Karwacz, K., Arce, F., Bricogne, C., Kochan, G., and Escors, D. (2012). PD-L1 co-stimulation, ligand-induced TCR down-modulation and anti-tumor immunotherapy. *Oncoimmunology* 1, 86–88

Karwacz, K., Bricogne, C., MacDonald, D., Arce, F., Bennett, C. L., Collins, M., et al. (2011). PD-L1 co-stimulation contributes to ligand-induced T cell receptor down-modulation on CD8+ T cells. *EMBO Mol Med* 3, 581–592

Khibnik, A. I., Kuznetsov, Y. A., Levitin, V. V., and Nikolaev, E. V. (1993). Continuation techniques and interactive software for bifurcation analysis of ODEs and iterated maps. *Physica D: Nonlinear Phenomena* 62, 360–371

Kim, D., Kwon, Y. K., and Cho, K. H. (2008). The biphasic behavior of incoherent feed-forward loops in biomolecular regulatory networks. *Bioessays* 30, 1204–1211

Kim, J. M. and Chen, D. S. (2016). Immune escape to PD-L1/PD-1 blockade: seven steps to success (or failure). *Ann. Oncol.*

Kindt, T. J., Goldsby, R. A., Osborne, B. A., and Kuby, J. (2007). *Kuby Immunology* (W.H.Freeman and Company, New York)

Kohlhapp, F. J., Huelsmann, E. J., Lacek, A. T., Schenkel, J. M., Lusciks, J., Broucek, J. R., et al. (2016). Non-oncogenic Acute Viral Infections Disrupt Anti-cancer Responses and Lead to Accelerated Cancer-Specific Host Death. *Cell Rep* 17, 957–965

Kosinsky, Y., Dovedi, S. J., Peskov, K., Voronova, V., Chu, L., Tomkinson, H., et al. (2018). Radiation and PD-(L)1 treatment combinations: immune response and dose optimization via a predictive systems model. *J Immunother Cancer* 6, 17

Kuznetsov, Y. A. (2013). *Elements of Applied Bifurcation Theory*, vol. 112 (Springer Science & Business Media)

Ledzewicz, U. and Schattler, H. (2017). Application of mathematical models to metronomic chemotherapy: What can be inferred from minimal parameterized models? *Cancer Lett.* 401, 74–80

Lever, M., Lim, H. S., Kruger, P., Nguyen, J., Trendel, N., Abu-Shah, E., et al. (2016). Architecture of a minimal signaling pathway explains the T-cell response to a 1 million-fold variation in antigen affinity and dose. *Proc. Natl. Acad. Sci. U.S.A.* 113, E6630–E6638

Lever, M., Maini, P. K., van der Merwe, P. A., and Dushek, O. (2014). Phenotypic models of T cell activation. *Nat. Rev. Immunol.* 14, 619–629

Levin, D., Forrest, S., Banerjee, S., Clay, C., Cannon, J., Moses, M., et al. (2016). A spatial model of the efficiency of T cell search in the influenza-infected lung. *J. Theor. Biol.* 398, 52–63

Liechtenstein, T., Dufait, I., Bricogne, C., Lanna, A., Pen, J., Breckpot, K., et al. (2012). PD-L1/PD-1 co-stimulation, a brake for T cell activation and a T cell differentiation signal. *J Clin Cell Immunol* S12

Liu, B., Chen, W., Evavold, B. D., and Zhu, C. (2014). Accumulation of dynamic catch bonds between TCR and agonist peptide-MHC triggers T cell signaling. *Cell* 157, 357–368

Marchuk, G. I. (1997). *Mathematical Modelling of Immune Response in Infectious Diseases*, vol. 395 (Springer Science & Business Media)

Marelli-Berg, F. M., Fu, H., Vianello, F., Tokoyoda, K., and Hamann, A. (2010). Memory T-cell trafficking: new directions for busy commuters. *Immunology* 130, 158–165

Martinez, M. R., Corradin, A., Klein, U., Alvarez, M. J., Toffolo, G. M., di Camillo, B., et al. (2012). Quantitative modeling of the terminal differentiation of B cells and mechanisms of lymphomagenesis. *Proc. Natl. Acad. Sci. U.S.A.* 109, 2672–2677

McKeithan, T. W. (1995). Kinetic proofreading in T-cell receptor signal transduction. *Proc. Natl. Acad. Sci. U.S.A.* 92, 5042–5046

Nirschl, C. J. and Drake, C. G. (2013). Molecular pathways: coexpression of immune checkpoint molecules: signaling pathways and implications for cancer immunotherapy. *Clin. Cancer Res.* 19, 4917–4924

Oelkrug, C. and Ramage, J. M. (2014). Enhancement of T cell recruitment and infiltration into tumours. *Clin. Exp. Immunol.* 178, 1–8

Ogawa, N., Ping, L., Zhenjun, L., Takada, Y., and Sugai, S. (2002). Involvement of the interferon-γ–induced T cell–attracting chemokines, interferon-γ–inducible 10-kd protein (CXCL10) and monokine induced by interferon-γ (CXCL9), in the salivary gland lesions of patients with Sjögren's syndrome. *Arthritis & Rheumatism* 46, 2730–2741

Ortega-Carrion, A. and Vicente-Manzanares, M. (2016). Concerning immune synapses: a spatiotemporal timeline. *F1000Research* 5

Parello, C. S. and Huseby, E. S. (2015). Indoctrinating T cells to attack pathogens through homeschooling. *Trends Immunol.* 36, 337–343

Pauken, K. E., Sammons, M. A., Odorizzi, P. M., Manne, S., Godec, J., Khan, O., et al. (2016). Epigenetic stability of exhausted T cells limits durability of reinvigoration by PD-1 blockade. *Science* 354, 1160–1165

Pauken, K. E. and Wherry, E. J. (2015a). Overcoming T cell exhaustion in infection and cancer. *Trends Immunol.* 36, 265–276

Pauken, K. E. and Wherry, E. J. (2015b). Snapshot: T cell exhaustion. *Cell* 163, 1038–1038

Poleszczuk, J. T., Luddy, K. A., Prokopiou, S., Robertson-Tessi, M., Moros, E. G., Fishman, M., et al. (2016). Abscopal Benefits of Localized Radiotherapy Depend on Activated T-cell Trafficking and Distribution between Metastatic Lesions. *Cancer Res.* 76, 1009–1018

Rendall, A. D. and Sontag, E. D. (2017). Multiple steady states and the form of response functions to antigen in a model for the initiation of T-cell activation. *R Soc Open Sci* 4, 170821

Richards, D. M., Kyewski, B., and Feuerer, M. (2016). Re-examining the nature and function of self-reactive T cells. *Trends Immunol.* 37, 114–125

Sakaguchi, S., Yamaguchi, T., Nomura, T., and Ono, M. (2008). Regulatory T cells and immune tolerance. *Cell* 133, 775–787

Schietinger, A. and Greenberg, P. D. (2014). Tolerance and exhaustion: defining mechanisms of T cell dysfunction. *Trends Immunol.* 35, 51–60

Sciammas, R., Li, Y., Warmflash, A., Song, Y., Dinner, A. R., and Singh, H. (2011). An incoherent regulatory network architecture that orchestrates B cell diversification in response to antigen signaling. *Mol. Syst. Biol.* 7, 495

Sharpe, A. H. and Pauken, K. E. (2017). The diverse functions of the PD1 inhibitory pathway. *Nat. Rev. Immunol.*

Simon, S. and Labarriere, N. (2017). PD-1 expression on tumor-specific T cells: Friend or foe for immunotherapy? *OncoImmunology* 0, 1–7

Sontag, E. D. (2013). *Mathematical Control Theory: Deterministic Finite Dimensional Systems*, vol. 6 (Springer)

Speiser, D. E., Ho, P. C., and Verdeil, G. (2016). Regulatory circuits of T cell function in cancer. *Nat. Rev. Immunol.* 16, 599–611

Spranger, S. (2016). Mechanisms of tumor escape in the context of the T-cell-inflamed and the non-T-cell-inflamed tumor microenvironment. *Int. Immunol.* 28, 383–391

Stein, J. V., Moalli, F., and Ackerknecht, M. (2016). Basic rules of T cell migration. In *Defects in T Cell Trafficking and Resistance to Cancer Immunotherapy* (Springer). 1–19

Tan, M. P., Gerry, A. B., Brewer, J. E., Melchiori, L., Bridgeman, J. S., Bennett, A. D., et al. (2015). T cell receptor binding affinity governs the functional profile of cancer-specific CD8+ T cells. *Clin. Exp. Immunol.* 180, 255–270

Tkach, K. and Altan-Bonnet, G. (2013). T cell responses to antigen: hasty proposals resolved through long engagements. *Curr. Opin. Immunol.* 25, 120–125

Toapanta, F. R. and Ross, T. M. (2009). Impaired immune responses in the lungs of aged mice following influenza infection. *Respir. Res.* 10, 112

Van Braeckel-Budimir, N. and Harty, J. T. (2017). Influenza-induced lung Trm: not all memories last forever. *Immunol. Cell Biol.* 95, 651–655

Vianello, F., Olszak, I. T., and Poznansky, M. C. (2005). Fugetaxis: active movement of leukocytes away from a chemokinetic agent. *J. Mol. Med.* 83, 752–763

Vonderheide, R. H. and June, C. H. (2014). Engineering T cells for cancer: our synthetic future. *Immunol. Rev.* 257, 7–13

Wang, C., Singer, M., and Anderson, A. C. (2017). Molecular dissection of CD8(+) T-cell dysfunction. *Trends Immunol.* 38, 567–576

Warmflash, A. and Dinner, A. R. (2009). Modeling gene regulatory networks for cell fate specification. *Statistical Mechanics of Cellular Systems and Processes* , 121

Wherry, E. J., Ha, S. J., Kaech, S. M., Haining, W. N., Sarkar, S., Kalia, V., et al. (2007). Molecular signature of CD8+ T cell exhaustion during chronic viral infection. *Immunity* 27, 670–684

Xie, J., Tato, C. M., and Davis, M. M. (2013). How the immune system talks to itself: the varied role of synapses. *Immunological reviews* 251, 65–79

Zhang, Y. and Rundell, A. (2006). Comparative study of parameter sensitivity analyses of the TCR-activated Erk-MAPK signalling pathway. *Syst Biol (Stevenage)* 153, 201–211

Zinselmeyer, B. H., Heydari, S., Sacristan, C., Nayak, D., Cammer, M., Herz, J., et al. (2013). PD-1 promotes immune exhaustion by inducing antiviral T cell motility paralysis. *J. Exp. Med.* 210, 757–774
